# Antibodies from primary humoral responses modulate the recruitment of naive B cells during secondary responses

**DOI:** 10.1016/j.immuni.2022.07.020

**Published:** 2022-10-11

**Authors:** Jeroen M.J. Tas, Ja-Hyun Koo, Ying-Cing Lin, Zhenfei Xie, Jon M. Steichen, Abigail M. Jackson, Blake M. Hauser, Xuesong Wang, Christopher A. Cottrell, Jonathan L. Torres, John E. Warner, Kathrin H. Kirsch, Stephanie R. Weldon, Bettina Groschel, Bartek Nogal, Gabriel Ozorowski, Sandhya Bangaru, Nicole Phelps, Yumiko Adachi, Saman Eskandarzadeh, Michael Kubitz, Dennis R. Burton, Daniel Lingwood, Aaron G. Schmidt, Usha Nair, Andrew B. Ward, William R. Schief, Facundo D. Batista

**Affiliations:** 1 The Ragon Institute of MGH, MIT, and Harvard University, Cambridge, MA 02139, USA; 2 Department of Immunology and Microbiology, The Scripps Research Institute, La Jolla, San Diego, CA 92037, USA; 3 IAVI Neutralizing Antibody Center, The Scripps Research Institute, La Jolla, San Diego, CA 92037, USA; 4 Center for HIV/AIDS Vaccine Development, The Scripps Research Institute, La Jolla, San Diego, CA 92037, USA; 5 Department of Integrative, Structural and Computational Biology, The Scripps Research Institute, La Jolla, San Diego, CA 92037, USA; 6 Department of Immunology, Harvard Medical School, Boston, MA 02115, USA; 7 Department of Microbiology, Harvard Medical School, Boston, MA 02115, USA

**Keywords:** antibody, humoral immunity, BG18, HIV, germinal center, SARS-CoV, RBD

## Abstract

Vaccines generate high-affinity antibodies by recruiting antigen-specific B cells to germinal centers (GCs), but the mechanisms governing the recruitment to GCs on secondary challenges remain unclear. Here, using preclinical SARS-CoV and HIV mouse models, we demonstrated that the antibodies elicited during primary humoral responses shaped the naive B cell recruitment to GCs during secondary exposures. The antibodies from primary responses could either enhance or, conversely, restrict the GC participation of naive B cells: broad-binding, low-affinity, and low-titer antibodies enhanced recruitment, whereas, by contrast, the high titers of high-affinity, mono-epitope-specific antibodies attenuated cognate naive B cell recruitment. Thus, the directionality and intensity of that effect was determined by antibody concentration, affinity, and epitope specificity. Circulating antibodies can, therefore, be important determinants of antigen immunogenicity. Future vaccines may need to overcome—or could, alternatively, leverage—the effects of circulating primary antibodies on subsequent naive B cell recruitment.

## Highlights

• Circulating antibodies affect the recruitment of naive B cells to germinal centers (GCs)
• Broad, low-affinity responses enhance naive B cells in GCs on secondary challenge
• High-titer, high-affinity, epitope-focused antibodies block cognate naive B cells
• Blocking can be overcome by the administration of excess antigen

## Graphical abstract

**Figure:**
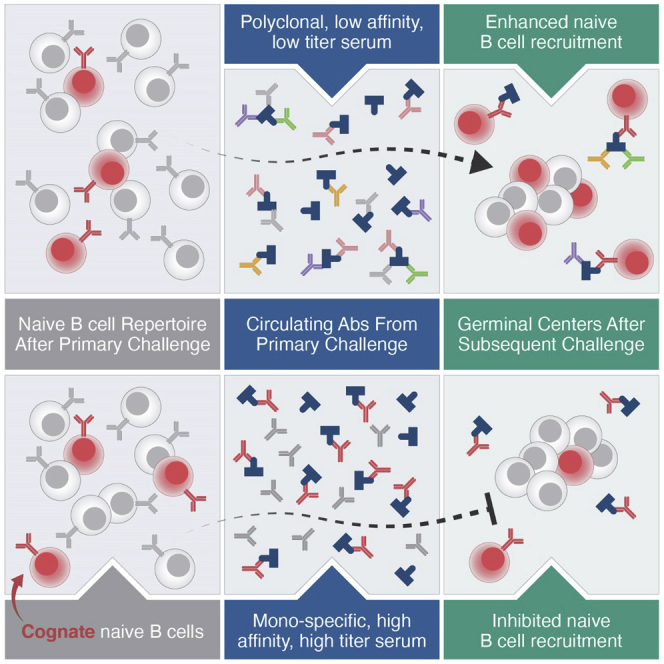

## Teaser

Using preclinical models for HIV and SARS-CoV, Tas et al. show that circulating antibodies from primary responses affect which naive B cells participate in germinal centers after secondary challenges. The directionality and intensity of this influence is determined by the epitope specificity of the primary response and by the affinity and concentration of the circulating antibody.

## Introduction

The generation of protective humoral immunity in response to antigen is a key feature of adaptive immunity. On initial antigen exposure, a clonally diverse pool of antigen-specific B cells is recruited to form germinal centers (GCs). Here, B cells undergo affinity maturation prior to taking up functions as antibody-secreting plasma cells (PCs) and memory B cells (MBCs) (Victora and Nussenzweig, 2022). Subsequent encounters with similar antigens induce increasingly potent secondary responses, in part due to experienced MBCs either rapidly differentiating into short-lived plasmablasts (PBs) or reengaging into secondary GCs (Gray, 1993; Kurosaki et al., 2015; Mesin et al., 2020; Weisel and Shlomchik, 2017). These anamnestic responses are fundamental to the efficacy of many vaccines, whereby the administration of one or successive doses of antigen is used to elicit protective serum antibody titers (Cyster and Allen, 2019; Pollard and Bijker, 2021). Consequently, the rules governing primary and secondary B cell responses—particularly the engagement of specific B cell populations—are of great interest to vaccine development.

The quality and diversity of the B cell clones that seed the initial GC are fundamental to a successful response, as recruiting cells that recognize diverse epitopes increases the likelihood of neutralizing pathogens (Scheid et al., 2009). Although early GCs tend to be diverse, the clones recruited to GCs are generally only a subset of the antigen-specific B cells in the total repertoire (Tas et al., 2016; Victora and Nussenzweig, 2022). Bias in B cell recruitment toward immunodominant epitopes could omit lesser-represented or lower-affinity clones, limiting, in turn, the opportunities to develop antibodies against other epitopes (Robbiani et al., 2020).

The recruitment of antigen-specific B cells to nascent GCs is in part governed by T cell help during the initial stages of the response; therefore, the ability of individual B cell clones to capture antigen and present their respective peptides to T cells is crucial (Schwickert et al., 2011; Yeh et al., 2018). The recognition and binding of B cells to an antigen is governed by a combination of physical properties, including epitope conformation, accessibility, and valency, as well as likeness to self-antigen (Bachmann et al., 1993; Batista and Neuberger, 1998; Kato et al., 2020; Kringelum et al., 2013). Many of these properties have been exploited to modulate the immunogenicity of vaccines and biological therapeutics (Bachmann and Jennings, 2010).

However, the humoral response against an antigen is not solely a function of these properties, as commensurate challenges can elicit varied responses (Laserson et al., 2014). Although genetic determinants may contribute (Briney et al., 2019), so does the immunological memory of past exposures (Auladell et al., 2022; Sallusto et al., 2010). The passive administration of antibodies has long been observed to variably enhance or inhibit downstream epitope-specific responses (Finkelstein and Uhr, 1964; Henry and Jerne, 1968; Heyman, 2000; Smith, 1909), and the term “original antigenic sin” was coined to describe how initial humoral responses to a pathogen can shape the outcome of subsequent encounters (Davenport et al., 1953; Francis, 1960; Zhang et al., 2019). In a salient recent example, a substantial part of the world population has been exposed to the SARS-CoV-2 virus through infection, vaccination, or both (Dong et al., 2020), and whether that exposure was via vaccination or natural infection shapes the response to subsequent infection (Röltgen et al., 2022).

The development of secondary GCs after subsequent exposures is primarily ascribed to naive B cell recruitment, as the GC participation of MBCs appears relatively limited and restricted to certain MBC subpopulations (Akkaya et al., 2020; Mesin et al., 2020; Pape and Jenkins, 2018b; Wong et al., 2020; Zabel et al., 2014). Conversely, however, antigen-specific serum, when transferred into a naive recipient, can block cognate naive B cell entry into GCs (Pape et al., 2011), and some MBCs do undergo further modification in GCs during secondary responses (McHeyzer-Williams et al., 2015). Clarifying secondary GC recruitment is of broad interest for rational vaccine design but may be particularly vital for HIV. Broadly neutralizing antibodies (bnAbs) against HIV, which bind conserved epitopes on the HIV envelope protein (Env), are central to current HIV vaccine research (Burton and Hangartner, 2016; Haynes and Mascola, 2017). To elicit mature bnAbs, a stepwise vaccine regimen to drive multiple rounds of affinity maturation against progressively more native-like immunogens has been proposed (Andrabi et al., 2018); the seeding of secondary GCs is, therefore, critical.

Here, we examined the recruitment of naive B cells to secondary GCs. Using preclinical SARS-CoV and HIV bnAb mouse models, we found that prior exposure established an epitope-specific affinity floor, mediated by circulating antibodies, directing the recruitment of specific cognate naive B cell populations. This is not necessarily deleterious: a broad polyclonal serum response to a SARS-CoV antigen favored the responses of underrepresented clones and enhanced the recruitment of higher-affinity clones during a secondary response; by contrast, however, epitope-specific high-affinity antibodies elicited by HIV bnAb priming immunogens attenuated epitope-specific naive B cell responses in a concentration-dependent fashion. Our findings on the circumstances under which peripheral antibodies inhibit or enhance naive B cell entry into GCs provide a conceptual framework for vaccine design.

## Results

### Primary humoral responses could impair the secondary recruitment of naive B cells

Current immunization strategies to elicit bnAbs to HIV rely on activating low-frequency precursors and shepherding them through sequential immunization (Burton and Hangartner, 2016; Jardine et al., 2013; Klein et al., 2013; Stamatatos et al., 2017; Steichenet al., 2019). We used a BG18 germline heavy-chain (HC) knockin (KI) precursor model (BG18^gH^) to investigate whether prior exposure to homologous antigen influenced naive bnAb precursor B cell participation in secondary responses. BG18 is a potent bnAb that targets an epitope comprising the conserved co-receptor sequence, GDIR, and a glycan at Asn^332^ (N332) (Barnes et al., 2018). Its germline-reverted HC was inserted into the murine IgH locus to create the BG18^gH^ mouse model in which 30%–32% of the B cell receptors (BCRs) consist of KI HCs paired with native murine light chains (LCs). BG18^gH^ was used to validate the engineered germline-targeting (GT) HIV Env-trimer N332-GT2 (geomean K_D_ of naive BG18^gH^ B cells: 582 nM; geomean K_D_ of sequenced human precursors: 519 nM) (Lin et al., 2018; Steichen et al., 2019).

**Figure 1:**
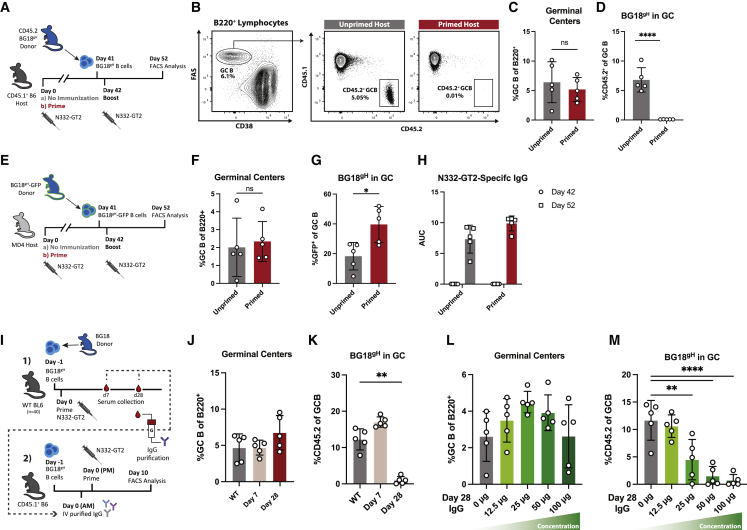
Previously elicited high-affinity antibodies restrict naive BG18^gH^ B cell responses (A) Schematic of evaluation of naive BH18^gH^ B cell responses to immunization by N332-GT2 NP in primed and unprimed recipients. (B) Representative FACS plots of gating strategy to quantify FAS^+^CD38^−^ GC B cells and CD45.2^+^ BG18^gH^ GC B cell responses versus endogenous CD45.1^+^ GC B cell responses in unprimed (left) and primed (right) recipients. (C and D) (C) GC cells as the percentage of total B cells and (D) CD45.2^+^ BG18^gH^ cells as the percentage of total GC B cells at experiment day 52 (10 days post-immunization [dpi]) in previously unprimed (gray) or primed (red) recipients. (E) Experimental design to evaluate naive BH18^gH^ B cell responses in primed and unprimed MD4 recipients. (F and G) (F) GC cells as the percentage of total B cells and (G) GFP^+^ BG18^gH^ cells as the percentage of total GC B cells at experiment day 52 (10 dpi) in previously unprimed (gray) or primed (red) MD4 BG18^gH^ recipients. (H) N332-GT2 trimer binding serum IgG of unprimed (gray) and primed (red) MD4 BG18^gH^ recipients at experiment day 42 (prior to immunization) and experiment day 52 (10 dpi). AUC = area under the ELISA binding curve. (I) Schematic of evaluation of the effect of serum IgG from previously immunized mice on naive BG18^gH^ recipients consisting of (1) isolation of serum from N332-GT2 immunized cohort and (2) administration of purified IgG to the naive BG18^gH^ recipients. (J and K) (J) GC cells as the percentage of total B cells and (K) CD45.2^+^ BG18^gH^ cells as the percentage of total GC B cells at 10 dpi in BG18^gH^ recipients receiving 200 μg of IgG isolated from 7- and 28-dpi serum. (L and M) (L) GC cells as the percentage of total B cells and (M) CD45.2^+^ BG18^gH^ cells as the percentage of total GC B cells at 10 dpi in BG18^gH^ recipients receiving escalating doses of IgG isolated from 28-dpi serum. p values were calculated by unpaired Student’s t test (C, D, F, and G) or ordinary one-way ANOVA with Dunnett’s multiple comparisons (K and M) (^∗^p < 0.05; ^∗∗^p < 0.01; ^∗∗∗∗^p < 0.0001; ns, not significant). Figures represent data from one of at least two experiments with 3–5 mice per condition, with data presented as mean ± SD. See also Figures S1–S3.

To investigate the effect of primary antigen exposures on secondary responses, naive CD45.2^+^ BG18^gH^ cells were adoptively transferred into CD45.1^+^ WT recipients to establish physiological precursor numbers (∼10–15 BG18^gH^ B cells per 10^6^ total mouse B cells). Recipients were either naive (unprimed) or previously immunized with N332-GT2 nanoparticle (NP) (primed); both groups were challenged with N332-GT2 NP 1 day after the transfer (Figure 1A). GC responses and the proportion of CD45.2^+^ BG18^gH^ GC B cells were evaluated 10 days post-immunization (dpi) (Figure 1B). Although the magnitudes of the GC responses were similar between both groups (mean splenic B cells: 5.2% in primed and 6.4% in unprimed), the participation of naive BG18^gH^ cells in the GC was reduced in primed hosts (mean 0.02% BG18^gH^ of the total GC B population in primed; 6.8% in unprimed) (Figures 1C and 1D). Additionally, BG18^gH^ B cells were similarly restricted during secondary responses when subjected to two consecutive homologous immunizations (prime and boost) (Figures S1A–S1C). The reduction in BG18^gH^ responses could indicate that the primary immune response to N332-GT2 NP limited the participation of these precursors.

To determine whether prior humoral response affected the secondary response, we investigated whether naive BG18^gH^ B cells were similarly restricted from GCs in mice unable to mount an effective humoral response against N332-GT2 NP. We used congenic MD4 mice in which humoral responses to antigens other than HEL and its homologs were severely compromised, as almost all mature B cells express a transgenic hen egg lysozyme (HEL)-specific BCR—although incomplete allelic exclusion in this model allows for a small number of WT BCRs (Mason et al., 1992). As both the BG18^gH^ and MD4 lines are CD45.2^+^, BG18^gH^ was crossed with an eGFP-expressing line to produce BG18^gH^-GFP. As above, a cohort of MD4 mice was primed with N332-GT2 NP, whereas another was left unprimed. Both MD4 cohorts were then adoptively transferred with naive BG18^gH^-GFP cells prior to immunization with N332-GT2 NP (Figures 1E and S2). Naive BG18^gH^-GFP cells were detected in GCs in both the primed (39.6% of the total GC B cells) and unprimed (21.7%) MD4 mice (Figures 1F and 1G), in contrast to the WT BG18^gH^ recipients above. The increase in BG18^gH^-GFP GC B cells in primed mice may have been due to low-affinity IgM elicited during the primary response, which could allow for improved immunogen sequestration and processing through immune complex (IC) formation (Heesters et al., 2014). Neither MD4 cohort had detectable N332-GT2-specific IgG titers on day 42, but both groups generated strong N332-GT2-specific IgG titers after the cell transfer and boost (Figure 1H). The contrast between the results of secondary immunization in WT and MD4 mice suggested that specific IgG responses contributed to BG18^gH^ restriction in WT.

To determine whether antibodies generated during primary immunization impair naive BG18^gH^ B cell responses, we investigated the effects of immunoglobulin isolated from primed mice in unprimed adoptive transfer recipients. A cohort of WT mice was adoptively transferred with BG18^gH^ cells and immunized with N332-GT2 NP a day later; serum was then collected on 7 and 28 dpi. Total IgG was purified using protein-G and administered to unprimed CD45.1^+^ mice (intravenously [i.v.], 200 μg/mouse) adoptively transferred with naive BG18^gH^ B cells the previous day. Approximately 4–6 h after receiving IgG, the CD45.1^+^ mice were immunized with N332-GT2 NP (Figure 1I). At 10 dpi, GC formation was similar between mice that received no serum and mice that received day-7 IgG; whereas there was a slight increase in GC size associated with day-28 IgG (Figure 1J). Meanwhile, there was a minor increase in CD45.2^+^ BG18^gH^ in GCs after the administration of day-7 IgG (mean 17.0% of GC B cells) over WT (12.2%), but CD45.2^+^ responses were strongly attenuated in mice receiving IgG isolated from day-28 serum (1.0%) (Figure 1K). As the affinity of BG18^gH^ B cells rapidly increases over time (Steichenet al., 2019), the curtailment by late time point IgG suggested that the affinity of the circulating antibodies could play a role.

To distinguish the effects of affinity and abundance, BG18^gH^ recipients were given incremental doses of the day-28 IgG isolates prior to immunization with N332-GT2 NP. N332-GT2-specific IgG serum titers prior to immunization corresponded closely with the dose of purified IgG received (Figure S3). In terms of GC size, we found an inflection point: up to 25 μg, an increase in antibody dose was associated with an increase in GC size; at higher concentrations, however, IgG was increasingly inhibitory (Figure 1L). By contrast, the BG18^gH^ GC responses diminished as mice received higher doses of purified IgG and were almost completely abrogated at the highest dose (0.7% CD45.2^+^ GC B cells at 100 μg IgG) (Figures 1L and 1M). Low affinities or low concentrations of circulating antibodies may have, therefore, enhanced the overall recruitment of B cells to GCs, whereas certain specific naive B cells may have been restricted at higher affinities and concentrations.

### Antibodies elicited during primary responses could, by contrast, enhance the recruitment of specific naive B cells

**Figure 2:**
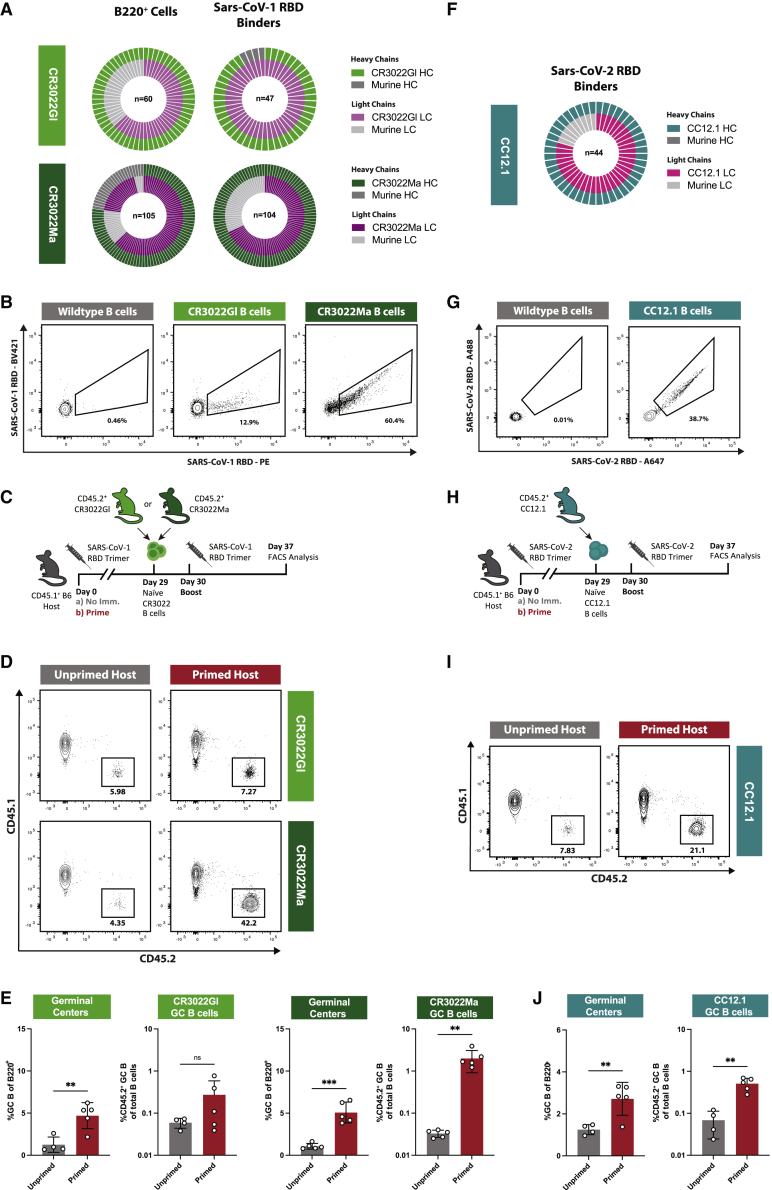
Naive CR3022Ma but not CR3022Gl B cell responses are enhanced in primed mice (A) (Top) Nested pie chart of CR3022Gl HC (light green), murine HC (dark gray), CR3022Gl LC (light purple), and murine LC (light gray) sequences amplified from single-cell sorted B220+ naive B cells from two CR3022GL mice. n = sequence pairs amplified. (Bottom) As top, for CR3022Ma. (B) Representative FACS plot of binding of naive CR3022Gl and CR3022Ma and WT B cells to SARS-CoV-1 RBD probes. (C) Schematic of evaluation of the effect of previous exposures on naive CR3022Gl and CR3022Ma B cell responses. (D) Representative FACS plots of gating strategy to quantify GC B cell responses as the percentage of total B cells and the percentage of CD45.2^+^ (here CR3022Gl/Ma) GC B cells of the total GC B cell population. (E) (Left) GC cells as the percentage of total B cells and (left-center) CR3022Gl GC B cells as percentage of total B cells at experiment day 40 (10 dpi) in previously unprimed (gray) or primed (red) CR3022Gl recipients. (Right-center) GC cells as the percentage of total B cells and (right) CR3022Ma GC B cells as the percentage of total B cells at experiment day 40 (10 dpi) in previously unprimed (gray) or primed (red) CR3022Ma recipients. (F) Nested pie chart of CC12.1 HC (teal), murine HC (dark gray), CC12.1 LC (pink), and murine LC (light gray) sequences amplified from single-cell sorted B220+ naive B cells from 2 CC12.1 mice. Center number indicates sequence pairs amplified. (G) Representative FACS plot of binding of naive CC12.1 and WT B cells to SARS-CoV-2 RBD probes. (H) Schematic of experimental evaluation of the effects of previous exposures on naive CC12.1 B cell responses in primed and unprimed recipients. (I) Representative FACS plots of gating strategy to quantify GC B cell responses as the percentage of total B cells and the percentage of CD45.2+ (here CC12.1) GC B cells of the total GC B cell population. (J) (Left) GC cells as the percentage of total B cells and (right) CC12.1 GC B cells as the percentage of total B cells at experiment day 37 (7 dpi) in previously unprimed (gray) or primed (red) CC12.1 recipients. p values were calculated by unpaired Student’s t test (^∗∗^p < 0.01; ^∗∗∗^p < 0.001; ns, not significant). Figures represent data from one experiment with 4–5 mice per condition, with data presented as mean ± SD. See also Figure S4.

We next sought to confirm these initial findings on naive B cell recruitment and GC size in an alternate preclinical vaccination model. To this end, BCR KI mice were generated based on a previously described broadly neutralizing SARS-CoV antibody, CR3022, and its inferred germline sequence (ter Meulen et al., 2006; Yuan et al., 2020). Briefly, using CRISPR-Cas9 as previously described (Lin et al., 2018; Wang et al., 2021a), mature, high-affinity CR3022 immunoglobulin (CR3022Ma) HC and variable LC regions, as well as germline-reverted, low-affinity CR3022 HC and LC (CR3022Gl), were inserted into the respective native loci. The resulting mouse lines expressed either CR3022Ma HC and LC or CR3022Gl HC and LC. B cells isolated from the peripheral blood of heterozygous HC^+/−^LC^+/−^ CR3022Gl and CR3022Ma animals expressed KI HC and LC almost exclusively (Figure 2A). These cells displayed high binding to SARS-CoV-1 RBD probes, indicating the expression of functional BCRs specific to SARS-CoV RBD (Figure 2B).

To determine whether antibody raised against SARS-CoV-1 RBD influenced the recruitment of naive KI B cells during secondary challenges, naive CD45.2^+^ CR3022Gl or CR3022Ma B cells were adoptively transferred into either WT CD45.1^+^ hosts (unprimed) or CD45.1^+^ hosts previously immunized (primed) with trimeric SARS-CoV-1 RBD (as described in Hauser et al., 2022) (Figure 2C). After receiving CR3022 B cells, the recipients were (re-)immunized with SARS-CoV-1 RBD trimer, after which GC responses and CD45.2^+^ CR3022 GC B cells were evaluated at 10 dpi (Figure 2D). Both high and low-affinity CR3022-bearing KI B cells participated in GCs in unprimed recipients (mean 6.1% and 3.2% of the total GC B for CR3022Gl and CR3022Ma, respectively) (Figure 2E). Priming produced a significant, ∼3-fold increase in the magnitude of GCs, with total GC B populations averaging 1.3% (unprimed) versus 4.7% (primed) in low-affinity CR3022Gl B cell recipients and 1.1% (unprimed) versus 5.1% (primed) in high-affinity CR3022Ma B cell recipients (Figure 2E). However, although we observed a minor increase in the number of low-affinity CR3022Gl GC B cells in primed recipients (mean 0.27% of the total B cells in primed versus 0.06% in unprimed), the proportion of high-affinity CR3022Ma GC B cells was more than fifty times higher in primed recipients (mean 2.0% of total B cells) than in unprimed recipients (0.03%) (Figure 2E). Rather than abrogating the recruitment to GCs, prior immunization enhanced the recruitment of high-affinity, but not low-affinity, precursors. Specific IgG titers were low immediately prior to boosting but increased in both groups of recipients after (Figure S4). We also generated a KI model for the mature SARS-CoV-2 antibody CC12.1 and used it to repeat these experiments (Figures 2F and 2G) (Rogers et al., 2020). We found a similar increase in CC12.1 GC B cells after RBD boost (Figures 2H–2J). These results and the differential responses to early and late N332-GT2 IgG observed above suggest that the affinities of both the circulating antibodies and the naive B cell population affect the seeding of secondary GCs.

### RBD responses were multi-epitope, whereas N332-GT2 responses were highly epitope focused

**Figure 3:**
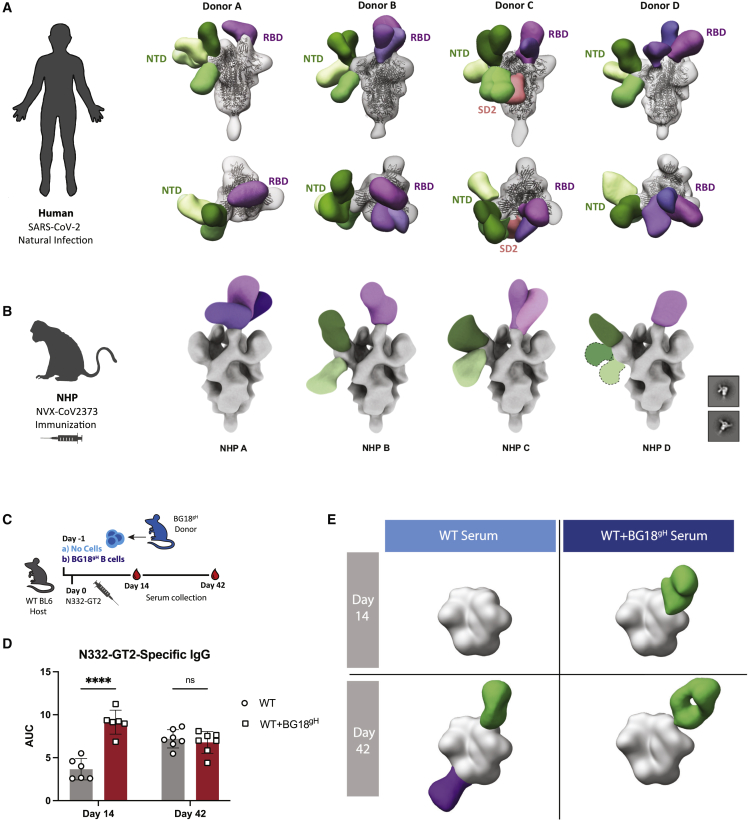
SARS-CoV spike responses engage multiple epitopes, whereas N332-GT2 responses are highly epitope focused (A) Summary of nsEMPEM analysis of human serum samples from four patients naturally infected with SARS-CoV-2. (B) Summary of nsEMPEM analysis of NHP serum samples from four subjects after immunization by NVX-CoV2373. (C) Schematic of experimental design to evaluate serum in WT and BG18^gH^-recipient mice 14 and 42 days post N332-GT2 immunization. (D) N332-GT2 binding serum IgG of WT B6 (gray) and BG18^gH^ recipients (WT+BG18^gH^, red) at 14 and 42 dpi. AUC = area under the ELISA binding curve. p values were calculated by unpaired Student’s t test (^∗∗∗∗^p < 0.0001; ns, not significant). Data from one experiment with 6–7 mice per condition and presented as mean ± SD. (E) Summary of nsEMPEM analysis of pooled mouse serum samples from (D). Composite models represent polyclonal antibody targeting against the N332-GT2 trimer. (Purple) Fabs targeting the base of the trimer; (green) Fabs targeting the V1/V3 region. See also Figure S5.

The differential effects on naive B cell in previously primed SARS-CoV and BG18^gH^ models led us to further dissect their primary IgG responses. First, we investigated serum antibody generated against natural SARS-CoV-2 infection in human donors by negative stain electron microscopy polyclonal mapping (nsEMPEM). SARS-CoV-2 infection elicited a range of primarily RBD and N-terminal domain (NTD)-directed antibodies, targeting diverse epitopes within these domains (Figure 3A). Additionally, antibodies from the serum of non-human primates (NHPs) immunized with NVX-CoV2373, a SARS-CoV-2 subunit vaccine using stabilized spike protein trimers (Dunkle et al., 2022), comparably targeted diverse NTD and RBD epitopes (Figure 3B). This is in line with the known diversity of convalescent and vaccine-induced SARS-CoV-2 antibodies (Ju et al., 2020; Wang et al., 2021b; Yuan et al., 2021).

Responses to HIV GT antigens, such as N332-GT2, are intended to be highly epitope focused to recruit rare bnAb precursors (Burton and Hangartner, 2016; Jardine et al., 2013; Klein et al., 2013; Stamatatos et al., 2017; Steichen et al., 2019). In N332-GT2-immunized WT mice and WT mice receiving BG18^gH^ B cells (Figure 3C), both endogenous murine B cells and BG18^gH^ B cells formed high titers of antibodies against the N332-GT2 trimer (Figure 3D). Although the BG18^gH^ model carries a diverse murine LC repertoire, many of these cells reach sub-nanomolar affinities against N332-GT2 within weeks following primary immunization (Steichen et al., 2019). This was illustrated by single particle electron microscopy (EM) analysis of murine polyclonal serum antibodies (Figure S5). We saw predominant binding against the BG18 epitope on N332-GT2 at both 14 and 42 dpi in the mice receiving BG18^gH^ B cells, as well as on day 42 in the WT sera, in addition to some endogenous base-directed responses (Figure 3E). This elicitation of a narrow epitope-specific subset of the B cell repertoire results in high-affinity responses to a single site, in contrast to the multi-site recognition induced by SARS-CoV-2 RBD.

### Monoclonal antibodies restricted BG18^gH^ B cells in a concentration-, affinity-, and epitope-dependent manner

**Figure 4:**
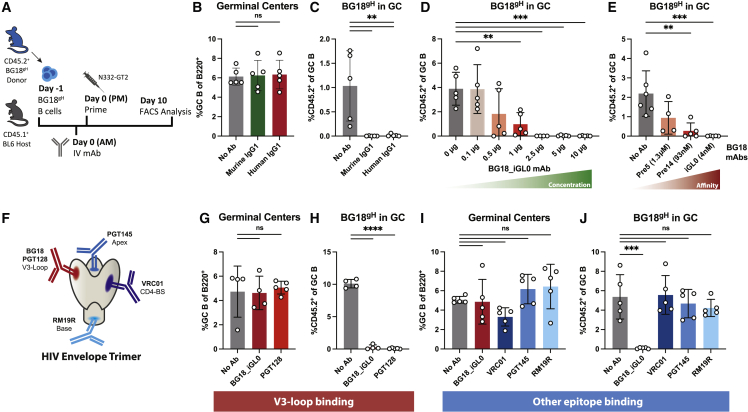
High-affinity mAbs restrict BG18^gH^ responses in an epitope-dependent manner (A) Schematic of experiment to evaluate the effects of i.v. administration of monoclonal antibodies on naive BH18^gH^ B cell responses in WT recipients. (B and C) (B) GC cells as the percentage of total B cells and (C) CD45.2^+^ BG18^gH^ cells as the percentage of total GC B cells at 10 dpi in BG18^gH^ recipients receiving 30 μg of either high-affinity murine IgG1 BG18 mAb (BG18_d42.10, green) or high-affinity human IgG1 BG18 mAb (BG18_iGL0, red). (D) CD45.2^+^ BG18^gH^ cells as the percentage of total GC B cells at 10 dpi of BG18^gH^ recipients receiving escalating concentrations of the high-affinity BG18_iGL0 mAb. (E) CD45.2^+^ BG18^gH^ cells as the percentage of total GC B cells at 10 dpi of BG18^gH^ recipients receiving 10 μg of BG18 mAbs with increasing affinity for the N332_GT2 immunogen (BG18_Pre5 with K_D_ 1.3μM, BG18_Pre14 with K_D_ 93 nM, and BG18_iGL0 with K_D_ 4 nM). (F) Schematic of the HIV Env trimer and binding sites of the bnAbs used in (G)–(J). (G and H) (G) GC cells as the percentage of total B cells and (H) CD45.2^+^ BG18^gH^ cells as the percentage of total GC B cells at 10 dpi in BG18^gH^ recipients receiving 10 μg of the BG18-epitope binding mAbs BG18_iGL0 and PGT128. (I and J) (I) GC cells as the percentage of total B cells and (J) CD45.2^+^ BG18^gH^ cells as the percentage of total GC B cells at 10 dpi in BG18^gH^ recipients receiving 10 μg of antibodies binding other epitopes on the N332-GT2 immunogen. p values were calculated by ordinary one-way ANOVA with Dunnett’s multiple comparisons (^∗^p < 0.05; ^∗∗^p < 0.01; ^∗∗∗^p < 0.001; ^∗∗∗∗^p < 0.0001; ns, not significant). Figures represent data from one of at least two experiments with 3–5 mice per condition, with data presented as mean ± SD.

To dissect the response to purified serum IgG, we sought to recapitulate it with monoclonal antibodies (mAbs). We administered mAbs with the same epitope specificity to the N332-GT2 molecule as the BG18^gH^ cells; naive BG18^gH^ B cell-recipient mice were given high-affinity N332-GT2-specific BG18-class mAbs i.v with either a murine or human IgG1 constant region (muBG18_d42.10 with K_D_ 0.5 nM or BG18_iGL0 with K_D_ 4.0 nM, respectively; Steichen et al., 2019) or no antibody (No Ab) and were then immunized with N332-GT2 NP (Figure 4A). Although the total GC responses remained roughly equivalent at ∼6% of B cells (Figure 4B), both murine and human IgG1 mAbs blocked the BG18^gH^ GC responses (Figure 4C). Similarly, increasing the dose of BG18_iGL0 decreased the BG18^gH^ GC responses, with near-complete abrogation above 2.5 μg (Figure 4D).

To further investigate the role of antibody affinity on the attenuation of the BG18^gH^ GC responses, mAbs with varying affinities for the BG18^gH^-epitope on N332-GT2 (BG18_iGL0 with K_D_ 4 nM, BG18_Pre14 with K_D_ 93 nM, and BG18_Pre5 with K_D_ 1.3 μM; Steichen et al., 2019) were administered i.v. to naive BG18^gH^-recipient mice prior to immunization. Although the high-affinity antibodies fully blocked the BG18^gH^ B cells from participating in the GC on day 10, lower-affinity antibodies allowed for some BG18^gH^ response (Figure 4E).

As both antibody titer and affinity for the antigen clearly played a role in the restriction of the BG18^gH^ GC responses, we next interrogated epitope specificity. Except for the modified BG18 binding site, the surface of the N332-GT2 trimer is mostly identical to that of the WT BG505 MD39 trimer (Steichen et al., 2016; Steichen et al, 2019), allowing other HIV bnAbs to bind their respective conserved epitopes on the molecule. We independently administered the overlapping V3-glycan antibody PGT128 (Pejchal et al., 2011; Walker et al., 2011), as well as antibodies binding more distal epitopes, including CD4-binding-site-directed VRC01 (Wu et al., 2010), apex-directed PGT145 (Lee et al., 2017; Walker et al., 2011), and MD39 base-directed RM19R (Cottrell et al., 2020), to naive BG18^gH^ B cell recipients prior to N332-GT2 NP immunization (Figure 4F). Administering PGT128, which overlaps with the mature BG18 epitope, prevented BG18^gH^ B cells from participating in the GC (Figures 4G and 4H). By contrast, none of the antibodies binding other epitopes on N332-GT2 excluded BG18^gH^ B cells from GCs (Figures 4I and 4J). Competition for identical or overlapping epitopes by circulating antibodies, thus, limited the response of cognate B cells to primary challenges, an inhibition that may also be relevant to secondary challenges.

### Circulating antibody affinity influenced VRC01-precursor B cell exclusion from GCs

**Figure 5:**
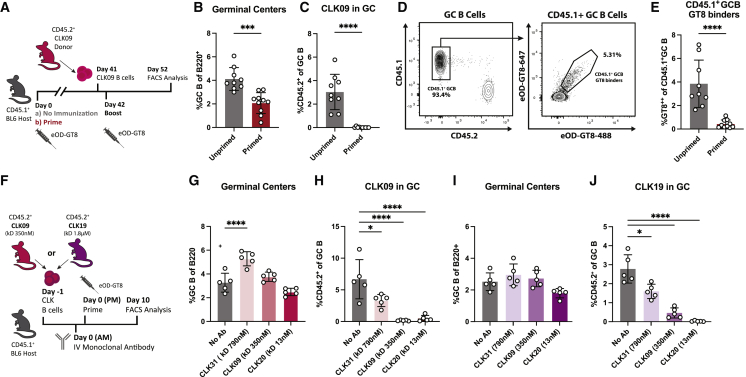
High-affinity antibody restricts VRC01-precursor responses **(**A) Schematic of experiment to evaluate naive CLK09 B cell responses in primed and unprimed recipients. (B and C) (B) GC cells as the percentage of total B cells and (C) CD45.2^+^ CLK09 cells as the percentage of total GC B cells at experiment day 52 (10 dpi) in previously unprimed (gray) or primed (red) recipients. (D) Representative FACS plots at experiment day 52 (10 dpi) of gating strategy to quantify eOD-GT8 binding in the endogenous CD45.1^+^ GC B cell population. (E) eOD-GT8 binding of endogenous CD45.1^+^ GC B cells at experiment day 52 (10 dpi) in unprimed and primed hosts. (F) Schematic of experiment to evaluate the effects of i.v. administration of 10 μg of CLK mAbs with increasing affinity for the eOD-GT8 immunogen (CLK31 with K_D_ 790 nM, CLK09 with K_D_ 350 nM, and CLK20 with K_D_ 13 nM) on naive CLK09 and CLK19 B cell responses in WT recipients (G–J). (G and H) (G) GC cells as the percentage of total B cells and (H) CD45.2^+^ CLK09 cells as the percentage of total GC B cells at 10 dpi of CLK09 recipients. (I and J) (I) GC cells as the percentage of total B cells and (J) CD45.2^+^ CLK19 cells as the percentage of total GC B cells at 10 dpi of CLK19 recipients. p values were calculated by unpaired Student’s t test (B, C, and E) or ordinary one-way ANOVA with Dunnett’s multiple comparisons (G–J) (^∗^p < 0.05; ^∗∗^p < 0.01; ^∗∗∗^p < 0.001; ^∗∗∗∗^p < 0.0001; ns, not significant). Figures represent data from one of at least two experiments with 3–5 mice per condition, with data presented as mean ± SD. See also Figure S6.

As BG18^gH^ KI germline HCs are paired with a variety of endogenous mouse LCs, the BG18^gH^ repertoire consists of a range of affinities for the N332-GT2 antigen (Lin et al., 2018; Steichen et al., 2019). To confirm our findings in an alternative bnAb precursor model and further delineate the role of BCR affinity, we turned to the previously generated VRC01 dual HC/LC KI germline precursor model, CLK09 (Wang et al., 2021a). CLK09 B cells bind the GT immunogen eOD-GT8 with a K_D_ of 350 nM and produce robust responses to primary immunization (Havenar-Daughton et al., 2018; Jardine et al., 2015; Wang et al., 2021a). CD45.2^+^ CLK09 cells were transferred into WT CD45.1^+^ recipients (∼10–15 precursors per 10^6^ total B cells) prior to immunization with eOD-GT8 60mer (Figure 5A). Similar to BG18^gH^ recipients, when naive CLK09 B cells were transferred into CD45.1^+^ hosts previously immunized with eOD-GT8 and subsequently given a second challenge, we found virtually no CLK09 B cells in the GC response 10 days post-secondary immunization (mean 3% versus 0.02% of GC B cells) (Figures 5B and 5C). Additionally, there was a marked reduction in the GC participation of endogenous eOD-GT8 binders (mean 3.8% versus 0.4% of CD45.1^+^ GC B cells) (Figures 5D and 5E). Thus, previously elicited antibodies restricted the clonality of primary responses from VRC01-class germline precursor B cells as well. There was also a reduction in CLK09 responses post-boost compared with post-prime (Figure S6), similar to BG18^gH^ (Figure S1). We hypothesize that antibody-mediated restriction of B cells will be important at all stages of highly epitope-focused humoral responses, although other aspects of the post-prime response, such as MBCs, may present confounding factors.

To investigate whether the relative difference in affinity between the BCR and competing antibody affects the dynamics of exclusion, we used B cells from both CLK09 and another VRC01-class germline precursor model, CLK19 (KI B cells from this line bind eOD-GT8 with a K_D_ of 1.8 μM), alongside a set of previously described CLK mAbs with a range of affinities for eOD-GT8 (Havenar-Daughton et al., 2018) (Figure 5F). When CLK09 B cell-recipient mice received mAbs of equal or higher affinity (CLK20: K_D_ 13 nM; CLK09: K_D_ 350 nM), the CLK09 GC response was almost completely blocked (mean 0.4% and 0.14% of GC B, respectively, versus 6.7% without mAb), whereas a lower-affinity mAb (CLK31: K_D_ 790 nM) did not fully block the response (3.3%) (Figures 5G and 5H). When mAbs were administered to CD45.1^+^ mice adoptively transferred with lower-affinity CLK19 B cells, however, the CLK31 mAb and the higher-affinity CLK09 mAb did not fully block the CLK19 GC B cell response (1.6% CLK19 GC B cells after CLK31 administration and 0.4% after CLK09, versus 2.8% without mAb) (Figures 5I and 5J). As the low-affinity precursors (CLK19) responds as well or better after high-affinity mAb administration than the high-affinity precursors (CLK09), the gap between mAb and BCR affinity does not map neatly to exclusion. Overall, however, a given clone’s participation was more thoroughly blocked by a higher-affinity mAb than a lower-affinity mAb, suggesting that circulating antibody affinity set a floor for GC entry.

### Antigen-specific antibodies only restricted corresponding B cells from GCs

**Figure 6:**
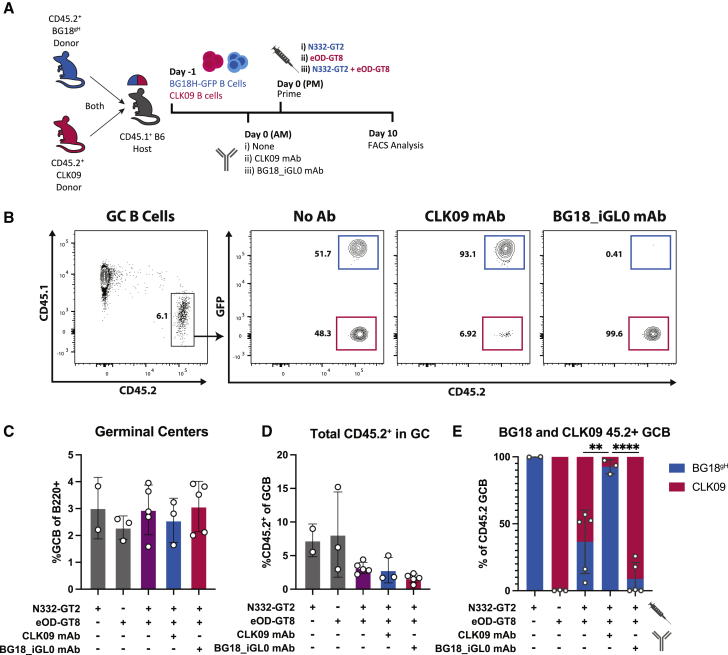
High-affinity antigen-specific antibody can restrict corresponding B cell populations from participating during co-evolution (A) Schematic of experiment to test the antibody-mediated restriction of immunogen-specific responses during co-evolution. (B) Representative FACS plots at 10 dpi of gating strategy to identify the effects of administration of no antibody, 10 μg of CLK09, or BG18_iGL0 mAb in BG18^gH^-GFP/CLK09 double recipients immunized with either N332-GT2 or eOD-GT8 or both (C–E). (C and D) (C) GC cells as the percentage of total B cells and (D) CD45.2^+^ population as the percentage of total GC B cells at 10 dpi. (E) Distribution of the CD45.2+ GC B cell populations between BG18^gH^-GFP (blue) and CLK09 (red) cells at 10 dpi. p values were calculated by unpaired Student’s t test (^∗∗^p < 0.01; ^∗∗∗∗^p < 0.0001). Figures represent data from one of at least two experiments with 2–5 mice per condition, with data presented as mean ± SD.

If different bnAb precursors respond to their respective antigens during the same GC response, it should be possible to restrict the participation of one but not the other B cell subset by administering the respective epitope-specific antibody. To test this, we transferred mixed CD45.2^+^ CLK09 and CD45.2^+^ BG18^gH^-GFP precursors into CD45.1^+^ mice, administered antigen-specific mAbs (CLK09 or BG18-iGL0), and challenged recipients with either one or both of their respective antigens (Figure 6A). GFP expression was used to distinguish the BG18^gH^-GFP and CLK09 CD45.2^+^ populations (Figure 6B). GCs were of similar magnitudes (Figure 6C), whereas CD45.2^+^ in GCs were somewhat decreased in recipients receiving both antigens (2.9%) relative to mice receiving a single antigen (7.8%), possibly due to increased competition with the endogenous repertoire (Figure 6D). Although individually administered immunogens recruited only their specific B cell populations to the GC, those that received both antigens had both BG18^gH^-GFP and CLK09 B cell populations in the CD45.2^+^ GCs. However, administering either BG18_iGL0 mAb or CLK09 mAb prior to immunization resulted in the exclusion of transferred B cells corresponding to the specificity of the mAb. After CLK09 mAb administration, 92.6% (mean, SD ± 5.2%) of the CD45.2^+^ population in the GC at 10 dpi consisted of BG18^gH^-GFP cells; while post BG18_iGL0 mAb administration, 91.8% (mean, SD ± 12.2%) of the CD45.2^+^ GC B cells were CLK09 (Figure 6E). These findings suggest that mAb administration could restrict individual epitope-specific precursors from participating in multivalent GC responses. Whether this approach to modulating clonal diversity is applicable when the non-desired epitope is present on the same protein, however, is unknown.

### Sequential antigen administration depleted peripheral antibody and allowed epitope-specific B cells to respond

**Figure 7:**
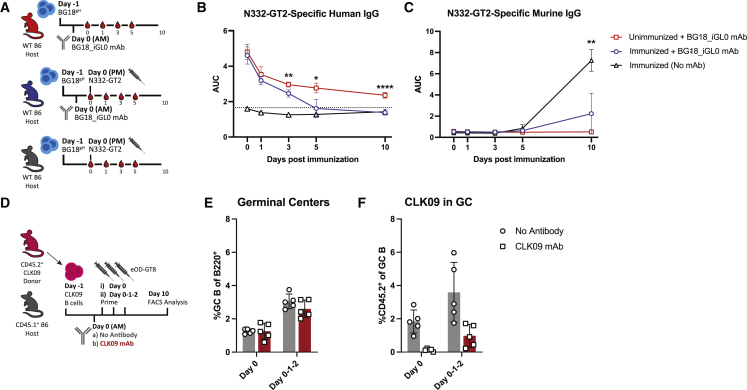
Excess antigen can overcome antibody-mediated restriction (A) Schematic of experiment to evaluate the antigen-mediated depletion of circulating antibodies. (B and C) (B) ELISA quantification of N332-GT2-specific human IgG and (C) N332-GT2-specifc murine IgG from mice receiving 10 μg BG18_iGL0 and subsequently immunized (blue) or left unimmunized (red) or mice that were immunized without receiving any mAb (black). p values were calculated by unpaired Student’s t tests comparing red versus blue (B) or blue versus black (C) groups at individual time points (^∗^p < 0.05; ^∗∗^p < 0.01; ^∗∗∗∗^p < 0.0001). (D) Schematic of experiment to test the effects of multiple doses of antigen on naive CLK09 B cell responses in CLK09 recipients receiving a single dose or triple doses of eOD-GT8 immunogen after receiving either 10 μg CLK09 mAb (red) or no mAb (gray) (E and F). (E and F) (E) GC cells as the percentage of total B cells and (F) CD45.2^+^ CLK09 cells as the percentage of total GC B cells at 10 dpi of CLK09 recipients. Figures represent data from one of at least two experiments with 3–5 mice per condition, with data presented as mean ± SD. See also Figure S7.

Our previous findings suggested a direct interaction between antigens and antigen-specific circulating antibody in the periphery. Circulating antibody is likely to rapidly bind available immunogen, so we expected immunogen-specific antibody titers to drop upon immunization. To investigate this, serum was collected in regular intervals from mice that had received BG18_iGL0 antibody prior to immunization (blue), as well as from control groups that either received no mAb prior to immunization (baseline, black) or received BG18_iGL0 mAb but were not subsequently immunized (natural decay, red) (Figure 7A). As BG18_iGL0 mAb has a human constant region, it could be measured independently from the endogenous mouse Ig responses. Over time, the curves show a drop in BG18_iGL0 antibody in mice receiving N332-GT2 NP, eventually declining to background levels at 10 dpi; the decay in unimmunized mice is far less severe (Figure 7B). The endogenous primary IgG response was diminished in the mice receiving BG18_iGL0, reflecting the restriction of BG18^gH^ response (Figure 7C). The relative drop in BG18_iGL0 suggests that antigen administration can deplete circulating antibody titers significantly; whether this would also be the case in a non-transfer model, where MBC-driven antibody production might overwhelm depletion, is unclear.

Based on these findings, we hypothesized that immunizing with excess immunogen over a longer period might overcome antibody blocking. To test this, a cohort of CLK09-recipient mice received a dose of CLK09 mAb followed by either a single immunization (day 0 group) or a series of three consecutive daily immunizations (day 0-1-2 group) (Figure 7D). Mice that received three immunizations had increased GCs in both conditions, likely due to the additional antigen providing a stronger immune activation potential (mean 1.3% GC B cells at single dose versus 2.8% at triple dose) (Figure 7E). We also observed that CD45.2^+^ cells in the GC were slightly increased in WT recipients receiving three doses of immunogen (mean 1.8% versus 3.6% of the total GC B cells). In mice receiving mAb prior to immunization, a single dose of antigen elicited little CLK09 response in GCs (0.12% of the total GC B cells), as previously observed. However, the administration of three doses slightly restored CLK09 GC responses (0.98%) (Figure 7F). The antibody-mediated restriction of B cell subsets might, therefore, be overcome through excess antigen, either by peripheral antibody depletion or an increased likelihood that some antigen escapes capture long enough to initiate a GC reaction.

As the BG18_iGL0 mAb is more potent than CLK09, we expected it to impose a more stringent barrier. In BG18^gH^ recipients receiving doses of either 2.5 μg BG18_iGL0 (the lowest blocking dose from Figure 4D), 10 μg BG18_iGL0, or No Ab, the GC formation in sequentially immunized mice were again enhanced compared with mice receiving only a single dose (mean 3.8% GC B at single dose versus 6.8% at triple doses) (Figures S7A and S7B). Despite this, the sequential administration of N332-GT2 was insufficient to overcome the restriction posed by 10 μg of BG18_iGL0 (Figure S7C). However, in recipients of 2.5 μg of BG18_iGL0, sequential immunization did elicit a small number of BG18^gH^ cells to the GC (mean 0.56% BG18^gH^ of the total GC B versus 4.2% without mAb) (Figure S7C). These findings illustrate that circulating antibody indeed posed a barrier, one that varied with affinity and concentration and could be overcome by repeat antigen administration.

## Discussion

The populating of secondary and ongoing GCs in response to secondary antigen challenges remains opaque (McHeyzer-Williams et al., 2018; Pape and Jenkins, 2018a; Shlomchik, 2018); it is clear, however, that naive B cells are the primary participants, at least in mice (Mesin et al., 2020). Here, we demonstrate that previously elicited antibodies, when at low titers or from broad, multi-epitope responses, enhanced naive the B cell recruitment to GCs. By contrast, high titers of high-affinity antibodies limited the participation of cognate naive B cells. This modulation by previously elicited antibodies has major implications for GC biology and vaccine design, particularly multi-dose vaccines.

The enhancement of naive B cell recruitment in mice previously exposed to SARS-CoV RBD is of urgent interest, considering the active development and deployment of booster regimens (Atmar et al., 2022; Gagne et al., 2022). Enhancement may result from multiple factors: previously generated antibodies can aid antigen transport and deposition through IC formation and complement fixation, functionally increasing the availability of antigen in the follicles (Batista and Harwood, 2009; Phan et al., 2009); this is likely what occurs after priming and the first boost, or during escalating doses (Tam et al., 2016). The lack of enhancement of off-target B cell recruitment after HIV mAb administration, however, complicates an IC-based interpretation of the SARS-CoV system. We expect that this difference reflects a lower rate of IC formation after mAb administration relative to polyclonal sera; the SARS-CoV RBD serum response is notably diverse, as shown in our infected human and immunized NHP data and by the range of SARS-CoV-2 antibodies in the literature (Ju et al., 2020; Wang et al., 2021b; Yuan et al., 2021). The affinity of serum antibodies is also likely to have a substantial role, as similar cognate naive cell recruitment enhancements were observed after the administration of early, low-affinity serum in the BG18 model and after priming the MD4 host. However, we would expect some of the antibodies in the SARS-CoV-2 response to be quite high affinity (Brouwer et al., 2020), so perhaps a combination of the low titers after only an antigen prime and the polyclonality of the circulating serum—which is higher for SARS-CoV-2 after vaccination than infection (Röltgen et al., 2022)—is the major source of enhancement in this system. However, our own models received a single prime: after repeated boosts, as the SARS-CoV-2 vaccine is currently deployed, increases in antibody affinity and titer could shift the balance toward inhibition. Such inhibition might be overcome by altering the SARS-CoV-2 spike protein in the vaccine—to Omicron BA.4, for example. New epitopes in variant spikes are unlikely to be recognized with high affinity by previously elicited antibodies and would, thus, be more likely to stimulate the recruitment of different naive cells into the GC. Relatedly, the antibody response to SARS-CoV-2 vaccines in patients who previously received mAb treatment is also likely be affected by epitope blocking.

The contrasting effects of previously elicited antibody in the HIV models demonstrated that the elicitation of quasi-mono-epitope high-affinity antibodies or passive administration of high-affinity, single-specificity mAbs inhibited the recruitment of certain antigen-specific naive B cells into GCs—although the effects on total GC size were mixed, and even the total exclusion of tracked cognate B cells did not prevent GC formation. Inhibitory effects for previously elicited antigen-specific antibodies have been observed before, although never dissected to the extent here. Passive administration of both IgG and IgM, for example, decreases GC volume or antigen-specific GC B cells (Bergström et al., 2017; Zhang et al., 2013). Immunoglobulin from hyperimmunized mice limits B cell responses to phycoerythrin and reduces responses from IgM^+^ MBCs in favor of responses from higher-affinity IgG memory subsets (Pape et al., 2011). The mechanism of such inhibition is still unclear; our mAb studies are suggestive of epitope masking, but as we also found increased antibody consumption following immunization, we suspect that epitope blocking and antibody-immunogen degradation may act cooperatively. Although this epitope-specific inhibition may appear to constitute a potential hurdle in the pursuit of bnAbs against HIV—which requires highly epitope-specific responses and multiple rounds of immunization—the alteration of the bnAb-epitope at each stage to create a heterologous, more native-like booster immunogens provides the opportunity to design immunogens with lower affinity for circulating serum antibodies (Steichen et al., 2016).

These observations raise the question of whether serum antibody feedback may overcome “antigenic sin” by driving diversification on secondary exposure. Previous serum studies, as well as *in silico* modeling, suggest that antibody feedback can shift humoral responses toward subdominant epitopes (Angeletti et al., 2017; Meyer-Hermann, 2019) and block GC B cell responses in an epitope-dependent manner (Forsell et al., 2017), presumably providing some counter to antigen seniority. On repeat immunization with the malaria circumsporozoite protein (CSP), as antibodies to the immunodominant repeats level out, responses are enhanced against subdominant epitopes (McNamara et al., 2020). Circulating antibodies focusing secondary responses toward epitopes not represented during the primary humoral response may, however, be detrimental during some recurring infections: more durable responses toward the conserved stalk domain of influenza hemagglutinin (HA) may be precluded in favor of strain-specific responses to the highly variable head domain in repeat influenza infections (Dreyfus et al., 2012; Ellebedy et al., 2020).

In terms of vaccination, there are several potential strategies to overcome antibody-mediated restriction in circumstances where it proves problematic. First, as we have done here, antigen availability can be addressed by increasing the amount of antigen administered; it has been previously demonstrated that escalating the doses of HIV gp120 improves antigen-specific humoral responses (Tam et al., 2016). Alternatively, the continuous delivery of immunogen through osmotic pumps or antigen deposition—which can increase the magnitude and diversity of B cell responses and resulting antibody titers (Hu et al., 2015; Tam et al., 2016)—could enhance availability. In line with our observations on epitope exclusion, these methods induce a shift away from the immunodominant epitopes typically favored during bolus immunization (Cirelli et al., 2019; Moyer et al., 2020). Increasing the time between doses to allow antibody titers to decline may also limit blocking, although decreased MBCs may be problematic for reengagement. Finally, a major opportunity could lie in new adjuvants, as many of those in use were selected for their ability to elicit sizable GCs and generate high circulating antibody titers (Pulendran et al., 2021). A separate class of adjuvants, with properties that prioritize MBC formation over high-affinity antibody titers, could be developed to deploy, for example, during HIV bnAb sequential immunization (Burton, 2019).

Ultimately, the elicitation of a productive antibody response depends on several factors, including, as demonstrated here, the affinity, abundance, and epitope specificity of the antibody in circulation. The interplay between B cells and the antibody environment may be one of the keys to the fine-tuned control of humoral responses.

### Limitations of the study

For several mAbs used, it is unknown whether binding is monovalent or bivalent. In a recent study, the unexpected inhibitory capacity of a non-overlapping, non-neutralizing, relatively low-affinity antibody to CSP is attributed to its avid binding (Vijayan et al., 2021), and we would expect this to be potentially relevant to our mAb experiments. Unfortunately, our attempts to explore this point with Fabs were limited by their short half-lives (data not shown).

Furthermore, the strength of a transfer system is that it allows for the isolation of desired cell populations. However, in natural secondary exposures, the primary immune response will have left more than circulating serum antibodies: interactions with other components of the primary humoral response could outweigh the effects of serum antibody. Additionally, although we have focused on naive B cell recruitment, some MBCs do reenter GCs (McHeyzer-Williams et al., 2018; Pape and Jenkins, 2018a; Shlomchik, 2018), and the effects of antibody feedback on potentially higher-affinity MBCs may shape the secondary humoral response in unexpected ways. For example, antibody feedback, in part on MBCs, can limit the response to vaccine boosters (McNamara et al., 2020). Finally, although we emphasize the biology of GC formation here, we did not track the fate of the transferred epitope-specific B cells outside of GCs.

## STAR★Methods

### Key resources table

**Table:** 

REAGENT or RESOURCE	SOURCE	IDENTIFIER
**Antibodies**		

Hamster monoclonal anti-mouse CD95 PE-Cy7 (clone: Jo2)	BD Biosciences	CAT#:557653
Rat monoclonal anti-mouse CD38 A700 (clone: 90)	Invitrogen	CAT#:56-0381-82
Mouse monoclonal anti-mouse CD45.2 PE (clone: 104)	Biolegend	CAT#:109808
Mouse monoclonal anti-mouse CD45.1 PerCP Cy5.5 (clone: A20)	Biolegend	CAT#:110728
Mouse monoclonal anti-mouse B220 A488 or BV421 (clone: RA3-6B2)	BD Biosciences	CAT#:557669, 562922
Rat monoclonal anti-mouse CD138 BV650 (clone:281-2)	BD Biosciences	CAT#:564068
Rat monoclonal anti-mouse IgD APC-Cy7	Biolegend	CAT#:405716
Rat monoclonal anti-mouse-CD16/32 purified (clone 2.4G2)	BD Biosciences	CAT#:553142
Rat monoclonal anti-mouse CD4 APC-eF780 (clone: RM4-5)	Invitrogen	CAT#:47-0042-80
Rat monoclonal anti-mouse CD8 APC-eF780 (clone: 53-6.7)	Invitrogen	CAT#:47-0081-80
Rat monoclonal anti-mouse F4/80 APC-eF780 (clone: BM8)	Invitrogen	CAT#:47-4801-80
Rat monoclonal anti-mouse Ly-6G APC-eF780 (clone: RB6-8C5)	Invitrogen	CAT#:47-5931-80
Monoclonal BG18_iGL0	Produced in house	N/A
Monoclonal BG18_Pre5	Produced in house	N/A
Monoclonal BG18_Pre14	Produced in house	N/A
Monoclonal muBG18_d42.10	Steichen et al., 2019	N/A
Monoclonal PGT128	Walker et al., 2011	AB_2491047
Monoclonal VRC01	Wu et al., 2010	AB_2491019
Monoclonal PGT145	Walker et al., 2011	AB_2491054
Monoclonal RM19R	Cottrell et al., 2020	N/A
Rabbit polyclonal anti-His	GeneScript	CAT#A00174
Human monoclonal CR3022 SARS-CoV-2 Spike Glycoprotein S1	Abcam	CAT#:ab273073
Alkaline Phosphatase AffiniPure Goat Anti-mouse IgG	Jackson Immuno Research	CAT#:115-055-071
Alkaline Phosphatase AffiniPure Goat Anti-human IgG	Jackson Immuno Research	CAT#:109-055-098

**Biological samples**		

Human plasma samples A (1988), B (1989) and C(1992) from Bangaru et al., 2022; Sample D from study PMID: 32540903	Bangaru et al., 2022; ongoing study	N/A
Immunized rhesus macaque serum	Novavax	N/A

**Chemicals, peptides, and recombinant proteins**		

Streptavidin-A488	Biolegend	CAT#:405235
Streptavidin-PE	Biolegend	CAT#:405204
Streptavidin-A647	Biolegend	CAT#:405237
Streptavidin-BV421	Biolegend	CAT#:405226
LIVE/DEAD™ Fixable Blue Dead Cell Stain Kit, for UV excitation	Thermo Fisher Scientific	CAT#: L34962
Sigma Adjuvant	Sigma	CAT#:S6322
Alhydrogel adjvant 2%	Invivogen	CAT#:Vac-alu-250
Papain	Sigma Aldrich	CAT #: P3125-100mg
N332-GT2 ferritin nanoparticle	Steichen et al., 2019	N/A
N332-GT2 trimer	Steichen et al., 2019	N/A
eOD-GT8 60mer immunogen	Jardine et al., 2015	N/A
SARS-CoV-1 RBD monomers	Produced in house	N/A
SARS-CoV-2 Spike Proteins	Produced in house	N/A
SIGMAFAST p-Nitrophenyl phosphate Tablets	Sigma	CAT#: N2770-50SET
Gentle Ag/Ab binding buffer	Pierce	CAT#:21012
Gentle Ag/Ab elution buffer	Pierce	CAT#:21013
ACK lysing buffer	Lonza	CAT#:10-548E
Monomeric and trimeric SARS-CoV-2 and SARS-CoV receptor binding domains	This paper	N/A
TALON Metal Affinity Resin	Takara	CAT#635652
ExpiFectamine 293 Transfection Kit	Thermo Fisher	Cat#A14525

**Critical commercial assays**		

Pan B Cell Isolation Kit II, mouse	Miltenyi Biotec	CAT#:130-104-443
UltraComp eBeads™ Compensation Beads	Thermo Fisher Scientific	CAT#:01-2222-42
Protein G chromatography cartridges, 5ml	Pierce	CAT#:89927
PD-10 desalting columns	GE Life Sciences	CAT#:17085101

**Deposited data**		

SARS-CoV-2 spike protein complexed with polyclonal Fab Human donor A Human donor B Human donor C Human donor D	This paper	EMDB: EMD-27200 EMDB: EMD-27199 EMDB: EMD-27198 EMDB: EMD-27197
S protein mix + NVX-vaccinated NHPs 36057 36964 37452 37611	This paper	EMDB: EMD-27039 EMDB: EMD-27040 EMDB: EMD-27041 EMDB: EMD-27042
N332-GT2 + Mouse Fab BG18, d14 WT, d42 BG18, d42	This paper	EMDB: EMD-27036 EMDB: EMD-27037 EMDB: EMD-27038
BCR sequences for CR3022Gl	This paper	GenBank: OP079065– OP079171; GenBank: OP079441–OP079547
BCR sequences for CR3022Ma	This paper	GenBank: OP079172–OP079380; GenBank: OP079548–OP079756
BCR sequences for CC12.1	This paper	GenBank: OP079381–OP079440; GenBank: OP079757–OP079826

**Experimental models: Cell lines**		

HEK-293T-F	Thermo Fisher Scientific	Cat#: R79007
Human: Expi293F	Thermo Fisher	Cat#A14527; RRID: CVCL_D615

**Experimental models: Organisms/strains**		

Mouse: B6.SJL-Ptprcapepcb/BoyJ	The Jackson Laboratory	JAX:002014
Mouse: C57BL/6J	The Jackson Laboratory	JAX:000664
Mouse : BG18gH	Lin et al., 2018	N/A
Mouse : BG18gH-GFP	This paper	N/A
Mouse : MD4	The Jackson Laboratory; Mason et al., 1992	JAX:002595
Mouse : CR3022Gl	This paper	N/A
Mouse CR3022Ma	This paper	N/A
Mouse CC12.1	This paper	N/A
Mouse : CLK09	Wang et al., 2021a	N/A
Mouse : CLK19	Wang et al., 2021a	N/A

**Oligonucleotides**		

sgRNA	Lin et al., 2018; Wang et al., 2021a	N/A

**Recombinant DNA**		

Plasmids for CR3022Gl, Ma and CC12.1	Produced in house	N/A

**Software and algorithms**		

FlowJo 10.8.1	Treestar	https://www.flowjo.com/
Prism 8.4.3	GraphPad	https://www.graphpad.com/
Leginon	Suloway et al., 2005	https://emg.nysbc.org/redmine/projects/leginon/wiki/Leginon_Homepage
Appion	Lander et al., 2009	N/A
DogPicker	Voss et al., 2009	N/A
RELION	Scheres et al., 2012	https://relion.readthedocs.io/en/release-3.1/
UCSF Chimera	Pettersen et al., 2004	https://www.cgl.ucsf.edu/chimera/

**Other**		

BD LSRfortessa	BD Biosciences	N/A
BD FACS ARIA II instrument	BD Biosciences	N/A
0.5 ml -10 kDa concentrator	Amicon Ultra	CAT #: UFC803096
15 ml-10 kDa concentrator	Amicon Ultra	CAT #: UFC901024
Protein G Sepharose 4 Fast Flow	Cytiva	CAT #: 17127903
AKTA pure system	Cytiva	N/A
FEI Tecnai Spirit T12 transmission electron microscope		N/A
TemCam F416 CMOS 4k x 4k camera	TVIPS	N/A
BioTek Synergy Neo2	Biotek	N/A
Biotek EL406 washer/dispenser	Biotek	N/A
Superdex 200 Increase 10/300 GL	GE Healthcare	Cytiva 28-9909-44

### Resource availability

#### Lead contact

Please direct requests for data and resources related to this study to lead contact Facundo D. Batista (fbatista1@mgh.harvard.edu).

#### Materials availability

Datasets and materials for this article are available from the lead contact on request under standard material transfer agreements.

### Experimental model and subject details

#### Mice

For immunization experiments, male B6.SJL-Ptprca Pepcb/BoyJ (CD45.1^+/+^), C57BL/6-Tg(IghelMD4)4Ccg/J (MD4^+^) and C57BL/6J mice between 8–12 weeks of age were acquired from The Jackson Laboratory (Bar Harbor, ME). CR3022Gl, CR3022Ma and CC12.1 KI lines were generated and initially bred at the animal facility of the Gene Modification Facility (Harvard University) and breeding for colony expansion and experimental procedures was subsequently performed at the Ragon Institute of MGH, MIT and Harvard. Previously generated BG18 germline HC KI mice (BG18^gH^), and VRC01 germline KI mice (CLK09, CLK19) were bred and maintained at the Ragon Institute of MGH, MIT and Harvard(Lin et al., 2018; Wang et al., 2021a). BG18^gH^ mice were crossed with C57BL/6-Tg(CAG-EGFP)131Osb/LeySopJ mice purchased from The Jackson Laboratory (Bar Harbor, ME) to generate BG18^gH^-GFP mice. All genotyping was done via ear or tail snips by TaqMan assay for a fee for service agreement (TransnetYX).

#### Human samples

Convalescent sera or plasma from four SARS-CoV-2 recovered human donors were used for EMPEM studies. Donors A, B and C correspond to donors 1988, 1989 and 1992 from our previous study (Bangaru et al., 2022); the studies were approved by the Institutional Review Board of Vanderbilt University Medical Center. Plasma from donor D was obtained from a different cohort of convalescent patients under protocol approved by the UCSD Human Research Protection Program (PMID: 32540903). The rhesus macaques (*Macaca mulatta*) were from Indian origin and sourced from Southwest National Primate Research Center (SNPRC) specific pathogen free colony and Envigo (Alice, TX). Sera from NVX-CoV2373-immunized NHPs were kindly provided by Novavax. The animals received 3 μg NVX-CoV2373 with 50 μg Matrix-M (Novavax AB, Uppsala, Sweden) spaced 21 days apart.

### Method details

#### Generation of CR3022Gl, CR3022Ma, and CC12.1 knockin (KI) mice

CR3022Gl, CR3022Ma and CC12.1 KI mice were generated following published protocols (Lin et al., 2018; Wang et al., 2021a). In brief, the targeting vector 4E10 (Ota et al., 2013) was modified by the incorporation of human rearranged VDJ (heavy chain construct) or VJ (light chain construct) sequences downstream of the promoter region and by elongation of the 5’ and 3’ homology regions using the Gibson assembly method (NEB). The targeting vector DNA was confirmed by Sanger sequencing (Eton Bioscience Inc.). Next, fertilized mouse oocytes were microinjected with a donor plasmid containing either the pre-rearranged IGH with the mouse V_H_J558 promoter, or the pre-rearranged IGK with the mouse Vκ4-53 promoter (200 ng/μl); two pair of single-guided RNAs (sgRNAs, 25 ng/μl) targeting either the H or the κ locus; and AltR-Cas9 protein (50 ng/μl) and injection buffer (Wang et al., 2021a). Following culture, resulting zygotes were implanted into the uteri of pseudopregnant surrogate C57BL/6J mothers.

#### SARS-CoV Immunogen and Flow Probe Protein Expression and Purification

The SARS-CoV-2 (Genbank MN975262.1) and SARS-CoV (Genbank ABD72970.1) spike protein receptor binding domains (RBDs) were used as the basis for constructing the trimeric SARS-CoV-2 and SARS-CoV immunogens and monomeric RBD flow probes as previously described (Hauser et al., 2022). Briefly, codon optimized constructs were purchased as gblocks from Integrated DNA Technologies and subsequently cloned into pVRC for expression. Monomeric constructs for flow cytometry contained C-terminal HRV 3C-cleavable 8xHis and Avi tags. Trimeric constructs included C-terminal HRV 3C-cleavable 8xHis tags and a non-cleavable hyperglycosylated GCN4 tag that included two engineered C-terminal cystines as a modification from a published hyperglycosylated GCN4 tag (Sliepen et al., 2015) as previously described (Hauser et al., 2022).

Proteins were expressed in Expi 293F cells (ThermoFisher) via transfections were performed with Expifectamine reagents per the manufacturer’s protocol. Transfections were harvested after 7 days, and supernatants were purified using Cobalt-TALON resin (Takara) for immobilized metal affinity chromatography via the 8xHis tag. Imidazole was used to elute proteins, which were then concentrated. Size exclusion chromatography with a Superdex 200 Increase 10/300 GL (GE Healthcare) column in PBS was used to further purify proteins.

#### Immunizations

For adoptive transfers, B cells were isolated from donor spleens via magnetic bead separation (Pan B cell isolation kit, Miltenyi Biotec), quantified for live cells (LUNA automated cell counter) and adjusted to desired concentration in sterile PBS prior to intravenous injection into recipient mice. For immunization of CR3022Ma and CR3022Gl mice, trimeric SARS-CoV-1 RBD as described above and in (Hauser et al., 2022), and prepared for immunization at 10 μg/mouse by diluting stock in sterile PBS and complexing at a 1:1 volume ratio with Sigma Adjuvant (Millipore Sigma). For immunization of all BG18^gH^ recipients, N332-GT2 ferritin nanoparticle was used (except for the epitope experiments where N332-GT2 trimer was used instead). Specifics and production of N332-GT2 immunogen was originally described in (Steichen et al., 2019). N332-GT2 immunogen was prepared for immunization at 5 μg/mouse by diluting N332-GT2 stock in sterile PBS and subsequently complexing it for 30 minutes at 4°C at 1:1 volume ratio with Sigma Adjuvant. The eOD-GT8 60-mer immunogen was also previously described (Abbott et al., 2018), and was similarly prepared for immunization at 15 μg/mouse by diluting eOD-GT8 stock in sterile PBS and complexing at a 1:1 volume ratio of Alhydrogel solution (Invivogen) for 30 minutes at 4°C. For experiments administering a mixture of both N332-GT2 and eOD-GT8 both antigens were diluted in sterile PBS to their respective concentrations prior to complexing with Sigma adjuvant. Prepared immunogen was subsequently injected intraperitoneally (IP) at a total volume of 200 μl/mouse. All animals were cared for in accordance with American Association for the Accreditation of Laboratory Animal Care (AAALAC) standards in accredited facilities. All animal procedures were performed according to protocols approved by the Institutional Animal Care and Use Committee (IACUC) of Harvard University and the Massachusetts General Hospital (MGH), specifically: Animal Study Protocols 2016N000022 and 2016N000286 (MGH).

#### Serum IgG purification and monoclonal antibody production

Serum was acquired from immunized mice via cheek bleed and subsequently pooled. The IgG from the pooled serum was then purified using a Pierce Protein G chromatography column (Thermo Fisher), and placed in PBS using a Centricon filter unit (EMD Millipore). Concentration was measured via A280 on a Synergy Neo2 instrument (Biotek) To produce monoclonal antibodies for administration to mice, genes encoding the antibody Fv regions were synthesized by GenScript and cloned into antibody expression vectors. Monoclonal antibodies were produced using transient transfection of HEK-293F cells (ThermoFisher) and purified using rProtein A Sepharose Fast Flow resin (Cytiva) or GammaBind G column (Cytiva) for the mouse antibody muBG18_d42.10.

#### Flow cytometry

At selected time points following immunization, whole spleens were mechanically dissociated to generate single-cell suspensions. ACK lysis buffer was used to deplete red blood cells, after which splenocytes were resuspended in FACS buffer (2% FBS in PBS), incubated with Fc-block (clone 2.4G2, BD Biosciences) and stained for viability with Live/Dead Blue viability dye (Thermo Fisher Scientific) for 20 min at 4°C. Cells were then stained with a variety of antibodies, including antibodies against CD4-APC-eF780, CD8-APC-eF780, Gr-1-APC-eF780, F4/80-APC-eF780, B220-V421, CD95-PE-Cy7, CD38-A700, CD45.1-PerCP-Cy5.5, CD45.2-PE, IgD-APC-Cy7, and C138-BV650. For surface staining of eOD-GT8 binding, probes were labeled as described previously and added 30 minutes prior to antibody staining (Wang et al., 2021a). Cells were acquired by a BD LSRFortessa (BD Biosciences) for flow cytometric analysis and sorted using a BD FACS Aria II instrument (BD Biosciences). Data was analyzed using FlowJo software (Tree Star).

#### ELISA

Antigen-specific antibody titers were detected by ELISA. N332-GT2 titers were detected using anti-His Ab (2 μg/ml) to capture N332-GT2 antigen (2 μg/ml) on 96- or 384-well high-absorption ELISA plates (NUNC/Corning). SARS-CoV-1 RBD specific titers were detected by coating 96-well high-absorbtion ELISA plates with SARS-CoV-1 RBD monomers (2.5μg/ml). Plates were then washed 3 times with 0.05% Tween 20 in PBS, blocked with 3% BSA in PBS for 1 h at room temperature (RT), and washed again prior to incubation with 1:100 serially diluted mouse serum samples for 1 hour at RT. Wells were washed and incubated with Alkaline Phosphatase AffiniPure Goat Anti-Mouse IgG (Jackson Immuno Research, #115-055-071) at 1:1,000 in PBS with 0.5% BSA for 1 h at RT for detection of mouse IgG. For detection of Human IgG Alkaline Phosphatase linked to AffiniPure Goat Anti-human IgG (Jackson Immuno Research, #109-055-098) was used at 1:1000. For detection, p-Nitrophenyl phosphate (Sigma, # N2770) was dissolved in ddH_2_O and added at 50 μl/well, RT, 25 min. Absorbance at 405 nm was determined with a plate reader (Synergy Neo2, BioTek). SARS-CoV-1 specific titers were measured through comparison to a CR3022Ma mAb standard. ELISA curves were analyzed and AUC/concentration calculated using GraphPad Prism 8.4.3 (GraphPad).

#### BCR sequencing

Following single-cell sorting of naïve or antigen-specific B cells, the genes encoding the variable region of the heavy and light chains of IgG were amplified through RT-PCR. In brief, first strand cDNA synthesis was carried out using SuperScript III Reverse Transcriptase (Invitrogen) according to manufacturer’s instructions. Nested PCR reactions consisting of PCR-1 and PCR-2 were performed as 25μl reactions using HotStarTaq enzyme (QIAGEN), 10 mM dNTPS (Thermo Fisher Scientific) and cocktails of IgG- and IgK-specific primers and thermocycling conditions described previously (von Boehmer et al., 2016). PCR products were run on precast E-Gels 96 2% with SYBR Safe (Thermo Fisher Scientific) and wells with bands of the correct size were submitted to GENEWIZ company for Sanger sequencing. HC products were sequenced using the HC reverse primer from PCR-2 (5’ GCTCAGGGAARTAGCCCTTGAC 3’) and the LC was sequenced using the LC reverse primer (5’ TGGGAAGATGGATACAGTT 3’) from PCR-2. Reads were quality-checked, trimmed, aligned, and analyzed using the Geneious software (Biomatters Ltd, New Zealand). IMGT/V-QUEST (http://www.imgt.org) was used for mouse/Human Ig gene assignments. Circleplots were generated in Graphpad Prism.

#### Expression and purification of SARS-CoV-2 spike proteins

A mixture of stabilized S proteins were used for nsEMPEM studies with SARS-CoV-2 spike. The base construct (SARS-CoV-2-HP) was generated with residues 1 to 1208 from the Wuhan-Hu-1 strain (GenBank: QHD43416.1) with six stabilizing proline (HexaPro) substitutions at positions 817, 892, 899, 942, 986, and 987 and the S1/S2 furin cleavage site modified to 682-GSAS-685 (Bangaru et al., 2022). The spikes were further modified to include D614G mutation and/or a combination of additional stabilizing mutations; Mut2 (S383C, D985C), Mut4 (A570C, L966C), and Mut7 (T883C, V705C). S protein expression and purification were carried out as in previously published methods (Bangaru et al., 2022).

#### Polyclonal Fab preparation from serum and sample preparation for electron microscopy

Isolation of IgG from serum and subsequent digestion to Fab was performed as previously described (Bianchi et al., 2018). For faster processing time and to observe the overall antibody response of the groups of mice used in this study, serum samples (0.5-1.0 mL serum volume range per animal) were pooled. Briefly, IgGs from mouse serum were isolated by a 3-day incubation with Protein G resin (Cytiva) at 4°C. IgGs were eluted with 0.1 M Glycine pH 2 and immediately neutralized with 1 M Tris pH 8. To prepare Fabs, IgGs were digested with liquid papain from papaya latex (Sigma Aldrich) for 5 hrs at 37°C and quenched with iodoacetamide. For higher throughput, size exclusion chromatography was not performed to separate the Fabs from papain, referred to as “dirty Fab”. For complexing, 15 μg of N332-GT2 trimer and 1 mg of “dirty Fab” were combined and incubated overnight at room temperature. The antigen-Fab complex was then size exclusion purified using a Superose 6 Increase (Cytiva) column to remove the excess Fabs and papain in the sample. The excess Fab peaks were pooled and stored at 4°C.

IgGs from NVX-CoV2373-immunized NHP sera and SARS-CoV-2 convalescent human sera were isolated in a similar manner with the use of Protein A and protein G resin (GE Healthcare), respectively. For polyclonal Fab preparation, IgGs were digested for 4-5 hr at 37°C with activated papain from papaya latex and quenched with 0.5M iodoacetamide followed by buffer exchanging to 1 x TBS (pH 7.4) using 10kDa centrifugal filter units. The digested Fab/Fc portion was purified over a Superose 200 increase 10/300 column (Cytiva) to remove any undigested IgG and concentrated using a 10kDa Amicon centrifugal filter unit (MilliporeSigma). For NHP samples, polyclonal Fab-spike complexes were made by incubating 250 μg of purified polyclonal Fabs with 15 μg of a mix of stabilized S proteins (SARS-CoV-2-6P-D614G, SARS-CoV-2-6P-Mut2-D614G, SARS-CoV-2-6P-Mut4-D614G, SARS-CoV-2-6P-Mut7-D614G, SARS-CoV-2-6P-Mut7). For human donor samples, complexes were made by incubating 5mgs of purified polyclonal Fabs with 20 μg of a mix of stabilized S proteins (SARS-CoV-2-HP, SARS-CoV-2-HP-Mut2, SARS-CoV-2-HP-Mut4 and SARS-CoV-2-HP-Mut7). Samples were incubated overnight at room temperature and complexes were purified over a Superose 6 increase 10/300 column (Cytiva) using UV 215 absorbance on an AKTA pure system (Cytiva). Protein fractions corresponding to the S protein were collected and concentrated using a 0.5-mL capacity 10kDa Amicon Ultra Centrifugal Filter Unit (MilliporeSigma).

#### Negative stain electron microscopy (nsEM)

Purified complexes were diluted to 0.03 mg/ml using 1X TBS pH 7.4, deposited on glow-discharged carbon coated copper mesh grids, and stained for 90 sec with 2% uranyl formate (w/v). Samples were imaged either on a FEI Tecnai Spirit T12 transmission electron microscope (120 keV, 52,000x mag, 2.06 Å per pixel, -1.5 μm defocus) equipped with an FEI Eagle 4k x 4k CCD camera or a FEI TF20 transmission electron microscope (200 keV, 62,000x mag, 1.78 Å per pixel, -1.5 μm defocus) equipped with a TVIPS TemCam F416 CMOS 4k x 4k camera. Collection of raw micrographs was automated with the Leginon (Suloway et al., 2005) software and stored in the Appion (Lander et al., 2009) database. For each mouse group complex, enough micrographs were collected to have ≥ 100k particles for data processing. Particles were picked using DogPicker (Voss et al., 2009), and further data processing was performed in RELION 3.0 (Scheres, 2012). For EMPEM with SARS-CoV-2 spike, an initial model was generated from a published SARS-CoV-2 S protein structure (PDB: 6VYB (Walls et al., 2020)) and used during data processing. Map interpretation and segmentation was performed in UCSF Chimera (Pettersen et al., 2004).

### Quantification and statistical analysis

For immunization studies, statistical analysis was performed in Prism 9.3.1 (GraphPad) using unpaired student’s t test assuming normal distributions, or one-way ANOVA test with Dunnett’s multiple comparisons. P values less than 0.05 were considered significant (^∗^p < 0.05; ^∗∗^p < 0.01; ^∗∗∗^p < 0.001; ^∗∗∗∗^p < 0.0001) as indicated in the figure legends.

## Data Availability

• Electron microscopy data for this article was deposited at the Electron Microscopy Data Bank (EMDB: https://www.ebi.ac.uk/emdb/) and are available as of the date of publication. EMD numbers are listed in the key resources table. BCR sequence data has been deposited to GenBank and is available as of the date of publication (https://www.ncbi.nlm.nih.gov/genbank/). Accession numbers are listed in the key resources table. • This paper does not report original code. • Any additional information required to reanalyze the data reported in this paper is available from the lead contact upon request.

## Supplemental information

Document S1. Figures S1–S7

Document S2. Article plus supplemental information
