# Supplementary material for: Antibodies from primary humoral responses modulate the recruitment of naive B cells during secondary responses
Source: Immunity. 2022 Oct 11;55(10):1856–1871.e6. doi: 10.1016/j.immuni.2022.07.020 (PMC9350677; doi:10.1016/j.immuni.2022.07.020)
Supplement: Document S1. Figures S1–S7 [file mmc1.pdf]

Immunity, Volume *55*

Supplemental information

Antibodies from primary humoral responses modulate the recruitment of naive B cells during secondary responses

Jeroen M.J. Tas, Ja-Hyun Koo, Ying-Cing Lin, Zhenfei Xie, Jon M. Steichen, Abigail M. Jackson, Blake M. Hauser, Xuesong Wang, Christopher A. Cottrell, Jonathan L. Torres, John E. Warner, Kathrin H. Kirsch, Stephanie R. Weldon, Bettina Groschel, Bartek Nogal, Gabriel Ozorowski, Sandhya Bangaru, Nicole Phelps, Yumiko Adachi, Saman Eskandarzadeh, Michael Kubitz, Dennis R. Burton, Daniel Lingwood, Aaron G. Schmidt, Usha Nair, Andrew B. Ward, William R. Schief, and Facundo D. Batista

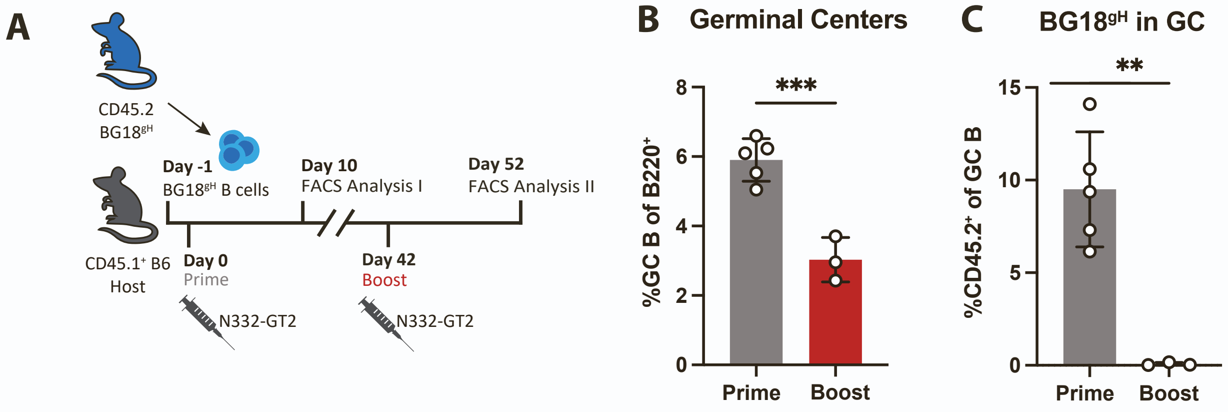

Figure S1. BG18^{gH} prime-boost response, related to Figure 1

A. Schematic representation of experimental design to compare the memory and naïve responses of BG18^{gH} B cells.

B. Quantification of GC cells as percentage of total B cells and **C.** Quantification of CD45.2^{+} BG18^{gH} cells as percentage of total GC B cells at experiment day 10 and 52 (10 days post primary and secondary immunization).

All p values were calculated by unpaired student's t test (** $p < 0.01$; *** $p < 0.001$).

Figures represent data from one of two experiments with 3-5 mice per condition. Data presented as mean ± SD

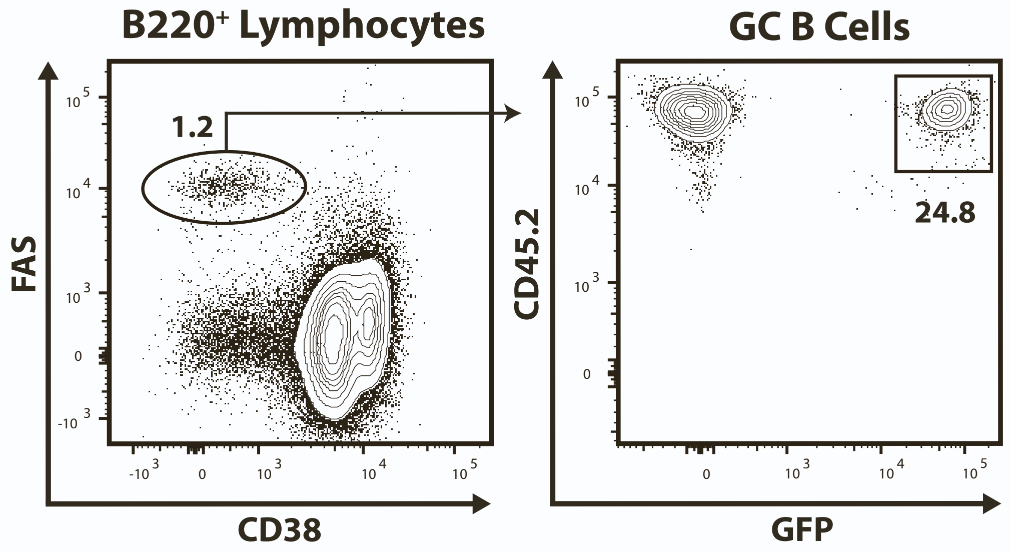

Figure S2. BG18gH-GFP gating strategy, related to Figure 1

Representative FACS plots at experiment day 52 (10 dpi) showing gating strategy to quantify FAS^{+}CD38^{-} GC B cells and GFP^{+} BG18^{gH} GC B cells.

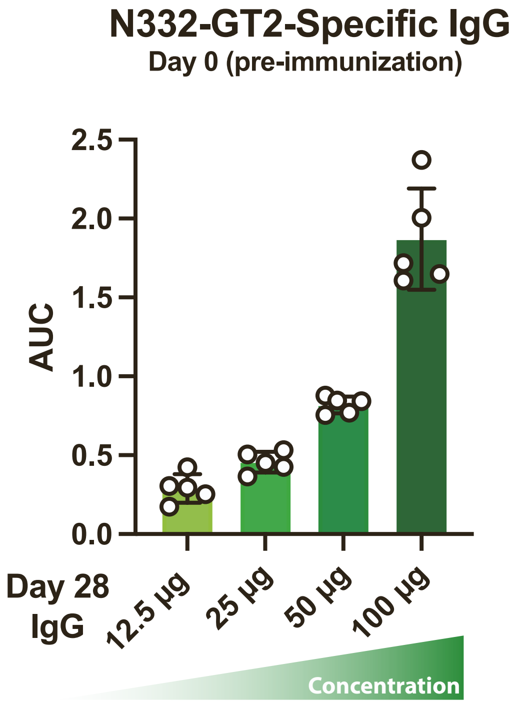

Figure S3. N332-GT2 specific IgG titers after transfer of isolated serum IgG, related to Figure 1

ELISA quantification of N332-GT2 binding serum IgG in naive recipients at day 0 after receiving intravenous serum IgG from previously immunized animal, prior to immunization. Data from one experiment with 5 mice per condition presented as mean ± SD

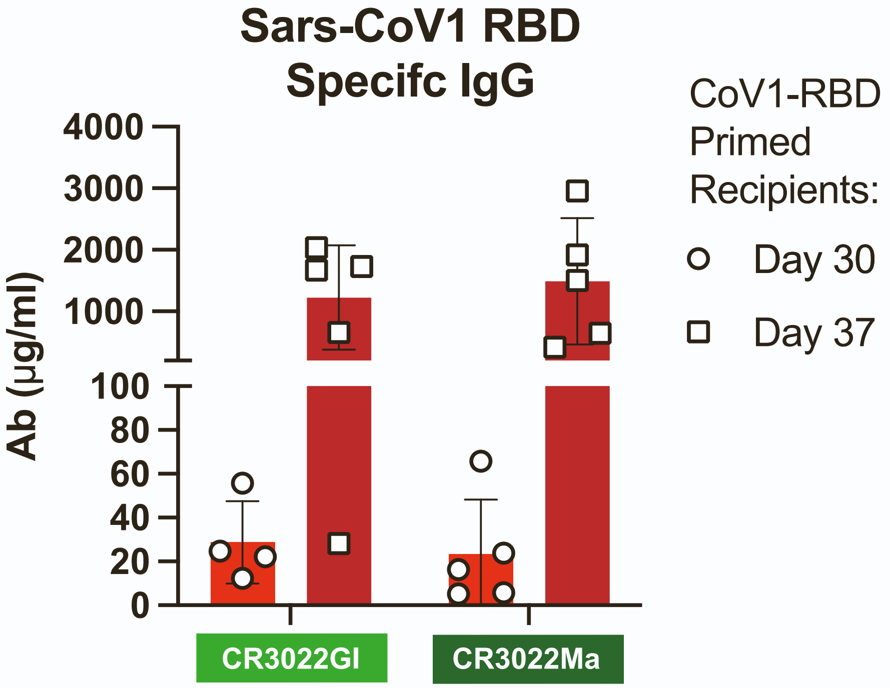

Figure S4. SARS-CoV-1 antibody titers in CR3022 recipients, related to Figure 2

SARS-CoV1-RBD-specific antibody titers determined by ELISA in primed CR3022Gl/Ma recipients pre- (day 30, light red) and post- (day 37, dark red) boost.

Data from one experiment with 4-5 mice per condition presented as mean ± SD

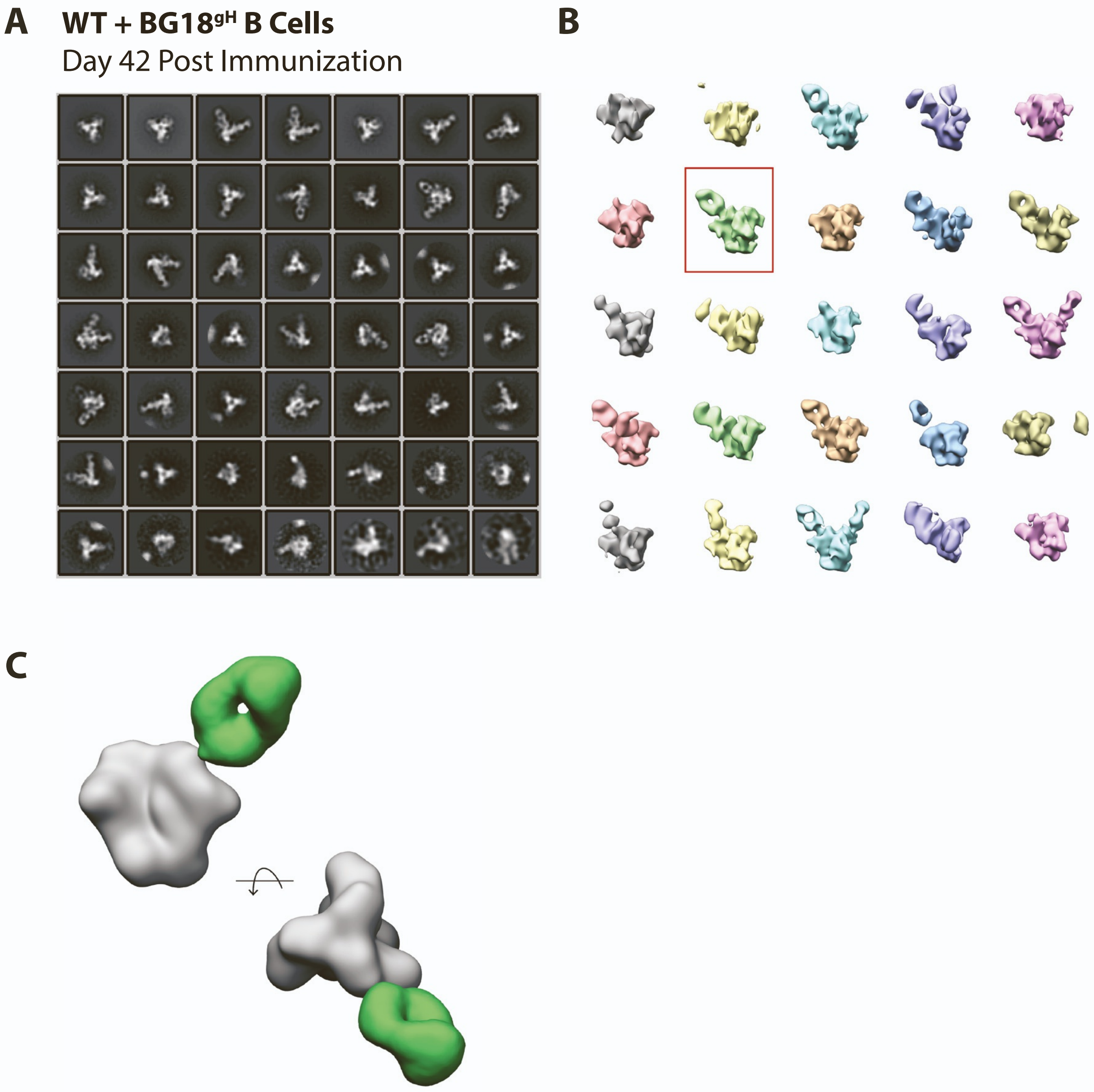

Figure S5. Data processing workflow for negative stain Electron Microscopy Based Polyclonal Epitope Mapping (nsEMPEM), related to Figure 3

A. Negative stain electron microscopy 2D class averages of polyclonal Fab from BG18 knock-in mice 42 days post immunization in complex with N332-GT2 trimer. **B.** Particles from the 2D class averages were sorted into 25 3D classes. Class 7 (boxed in red) was selected for refinement.

C. Composite model representing the refined Fab from Class 7 in complex with the N332-GT2 trimer.

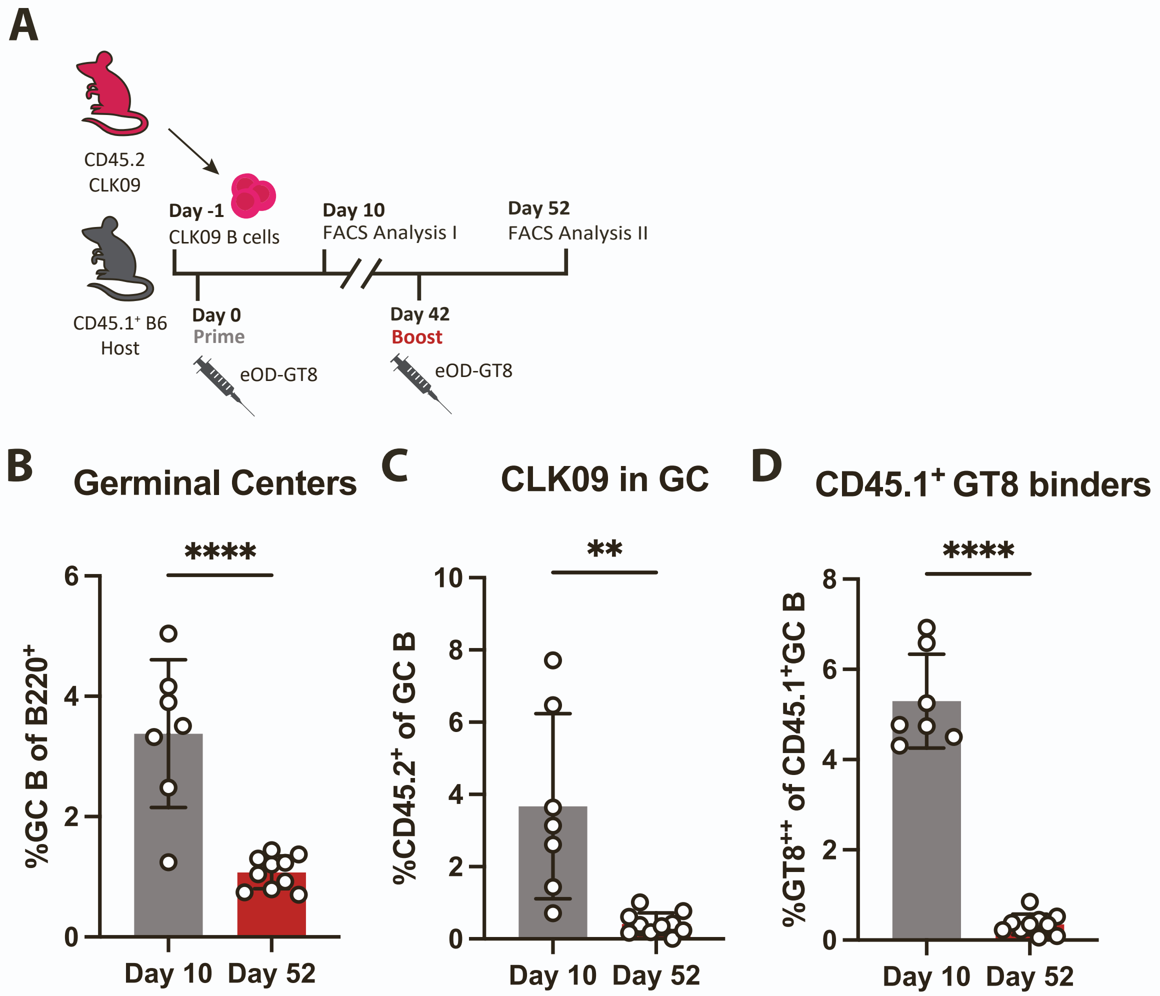

Figure S6. CLK09 prime-boost response, related to Figure 5

A. Schematic representation of experimental design to compare the memory and naïve responses of CLK09 B cells.

B. Quantification of GC cells as percentage of total B cells and **C.** Quantification of $CD45.2^{+}$ CLK09 cells as percentage of total GC B cells at experiment day 10 and 52 (10 days post primary and secondary immunization).

D. Quantification of eOD-GT8 binding endogenous $CD45.1^{+}$ GC B cells plots at experiment day 10 and day 52 (10 days post prime/boost).

All p values were calculated by unpaired student's t test (** $p < 0.01$; **** $p < 0.0001$).

Data from one experiment with 7-10 mice per condition presented as mean ± SD

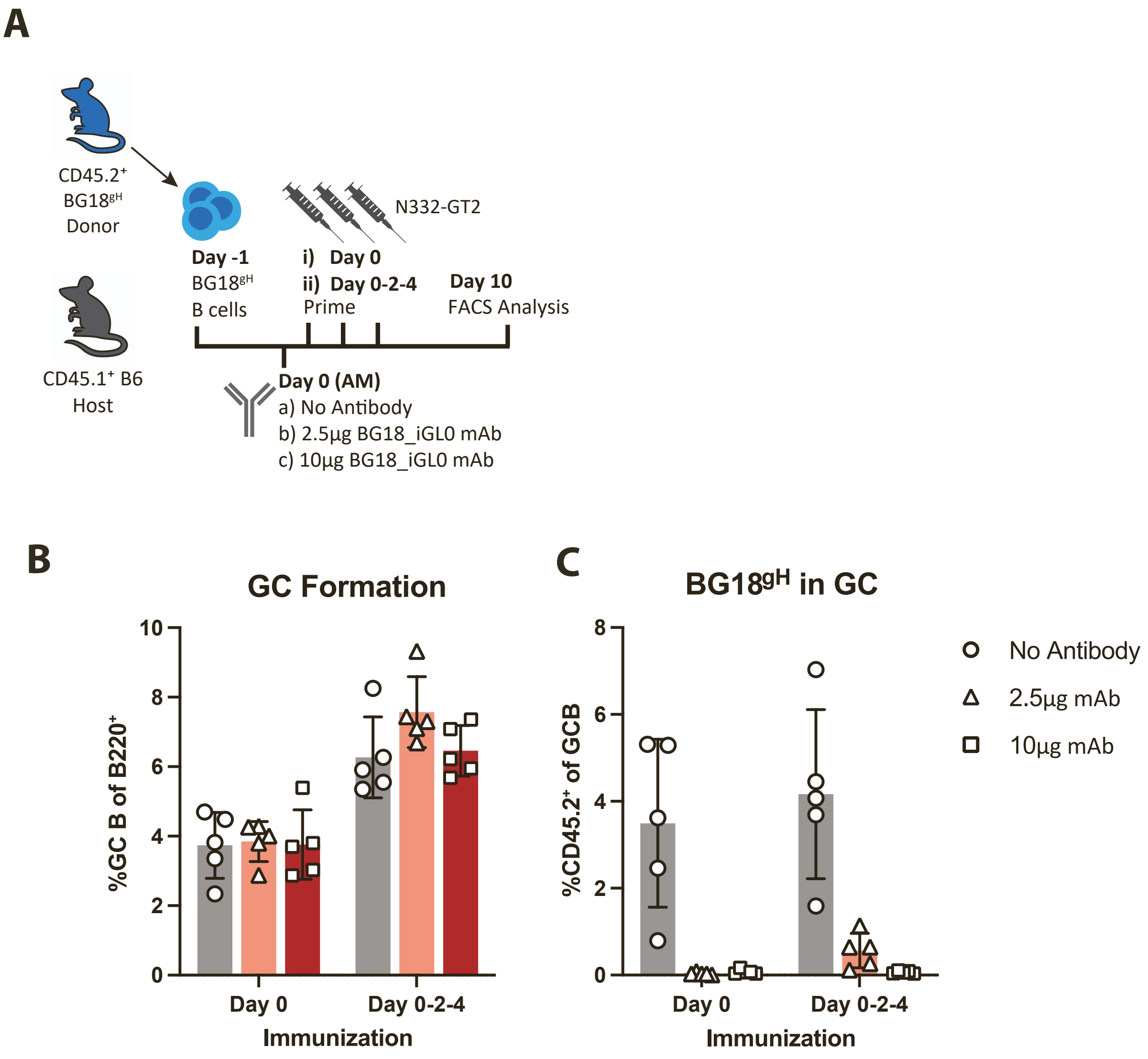

Figure S7. Response to sequential immunization with and without BG18_iGL0 mAb, related to Figure 7

A. Schematic representation of experimental design to test the effects of multiple doses of antigen on naïve BG18^{gH} B cell responses in BG18^{gH} recipients with or without mAb.

B Quantification of GC cells as percentage of total B cells and **C.** Quantification of CD45.2^{+} BG18^{gH} cells as percentage of total GC B cells at 10 dpi of BG18^{gH} recipients receiving a single or triple doses of N332-GT2 immunogen after receiving either 10µg BG18_iGL0 mAb (red), 2.5µg BG18_iGL0 mAb (orange) or no mAb (grey).

Data from one experiment with 5 mice per condition presented as mean ± SD
